# Testing of pandemic ventilators under early and agile development

**DOI:** 10.3389/fmedt.2022.899328

**Published:** 2022-08-16

**Authors:** Nikolaos Tachatos, Nicola Steffen, Mark Zander, Nikola Stankovic, Mirko Meboldt, Thomas O. Erb, Jürg Hammer, Marianne Schmid Daners

**Affiliations:** ^1^Product Development Group Zurich, Department of Mechanical and Process Engineering, ETH Zürich, Zurich, Switzerland; ^2^Department of Anesthesiology, University Children's Hospital Basel, University of Basel, Basel, Switzerland; ^3^Division of Respiratory and Critical Care Medicine, University Children's Hospital Basel, University of Basel, Basel, Switzerland

**Keywords:** respiratory devices, standardized testing, test protocol, *in vitro* testing, SARS-CoV-2, COVID-19, pandemic ventilator

## Abstract

Aiming to address clinical requirements subsequent to SARS-CoV-2-related pulmonary disease, multiple research groups and industry groups carried out intensive studies to develop pandemic ventilators (PDVs). *In vitro* testing to critically evaluate the specific performance of the developed apparatuses is an essential requirement. This study presents a test protocol which promotes a test-oriented, iterative, and agile assessment and consecutive development of such PDVs. It allows for fast identification of specific characteristics of each PDV in the individual test features. The test protocol includes an evaluation of the accuracy of control systems and instruments at changing parameters, the oxygen dynamics, and the response to trigger signals. The test environment is a mechanical lung, which allows reproducing various lung mechanics and to simulate active breathing cycles. A total of three PDVs that are under development were iteratively tested, with a Hamilton T1 as a reference. Continuous testing of the PDVs under development enables quick identification of critical application aspects that deserve further improved. Based on the present test protocol, the ventilators demonstrate a promising performance justifying continued development.

## Introduction

The SARS-CoV-2 pandemic caused an immediate shortage of mechanical ventilators and trained medical personnel to treat critically ill patients suffering from respiratory failure (1–3). Numerous research groups initiated intensive efforts to develop pandemic ventilators (PDVs) that are cost-efficient, simple to use, and fast to produce (3, 4).

The Medicines and Healthcare Products Regulatory Agency (MHRA) of the British government published a specification of the “minimally clinically acceptable” ventilator to be used in U.K. hospitals to treat patients requiring invasive ventilation due to respiratory failure caused by SARS-CoV-2 (5). These specifications describe minimal requirements for the hardware, the operational modes, the usability of alarms, and the safety features of PDVs.

In this context, a systematic test approach seems essential to support the development and fast-track assessment of low-cost PDVs. As of today, various teams have performed *in vitro* tests of their PDVs (6–11). We established a process to iteratively test the performance and features of PDVs and to provide further guidance in the early development phase. The protocol covers parts of the norm ISO 80601-2-12:2020 (12) and the MHRA recommendation (5). It is mainly designed to allow fast assessment of the different ventilator characteristics and features with an automated evaluation procedure. The protocol includes (a) a defined test procedure to evaluate the performance of the PDVs, (b) the use of a mechanical lung system that can mimic basic lung mechanics, and (c) the use of a reference commercial high-end ventilator for comparison. This study describes the methodology of an iterative test-oriented development based on this protocol. In addition, our test process is used to iteratively assess the performance of two pressure-controlled and one volume-controlled PDV which are under development.

## Materials and methods

### Ventilators

In this study, the protocol was applied for testing three novel PDVs and one clinical approved ventilator as reference: the GirVent (Girtec AG, Nänikon, Switzerland), the High Energy Ventilator (HEV, CERN, Geneva, Switzerland) (8), and the breathe (ETH, Zurich, Switzerland) (9). The three PDVs selected represent the three different most commonly used functional principles. Each PDV tested in this study is developed by the corresponding team and is still under development. The test status presented here dates from December 2020. The GirVent is a pressure-controlled ventilator based on a turbine blower with two oxygen (O_2_) ports: one port is realized by a direct injection of O_2_ into the patient tubing (LP1), while the other includes a 2-L balloon as reservoir (LP2). The HEV ventilator is based on a controlled valve system with a buffer reservoir in which the O_2_ concentration of the supplied air can be adjusted. The volume-controlled breathe ventilator (13) is built on the concept of the MIT E-Vent (14) and consists of a resuscitator bag (AmbuBag, Ambu, Ballerup, Denmark), which is periodically compressed by two paddles. The Hamilton T1 (Hamilton, Bonaduz, Switzerland) was included as a reference ventilator and was tested using the same test protocol as the PDVs.

Table 1 depicts the relevant features of the ventilators tested in this study. Each ventilator system was tested using the intended auxiliary materials supplied by the developer, that is, tubing, sensors, high-efficiency particulate air filters, and PEEP valves. The ventilation modes tested were pressure-controlled continuous mandatory ventilation (PC-CMV) and volume-controlled continuous mandatory ventilation (VC-CMV). For the Hamilton T1, the volume-targeted (S)CMV+ and pressure-controlled PCV+ mode was tested. In each ventilator system, the flow and pressure sensors are placed at the distal end of the patient tubing. The breathe and GirVent ventilators do not have an O_2_ sensor and do not include a closed-loop FiO_2_ control system. Both ventilators operate with a low-pressure O_2_ supply in feed-forward settings where the O_2_ flow is adjusted directly on the O_2_ source. The HEV ventilator operates with a high-pressure O_2_ supply and comprises a sensor that measures the O_2_ concentration in the buffer reservoir. Hence, in the HEV, FiO_2_ can be controlled in a closed loop similarly to the Hamilton T1. The Hamilton T1 can be operated with a low- (feed-forward) or a high-pressure (closed-loop) supply.

**Table 1:** Overview of the basic characteristics and auxiliary material of each ventilator tested.

**Ventilator**	**Modes tested**	**Ventilation mechanism**	**Flow and pressure sensor**	**PEEP mechanism**	**O_2_ control**	**Tubing system**	**Trigger detection**
Girtec GirVent	PC-CMV	Blower	In house development	Active by blower	Low-pressure	Double lumen	Pressure
CERN HEV	PC-CMV	Pressurized valve system	Hamilton flow and pressure sensor	Passive valve	High-pressure	Coaxial tube	Flow
ETH Zurich breathe	VC-CMV	Resuscitator	SFM3019 and MFPX5010DP	Passive valve	Low-pressure	Single lumen tube	Flow
Hamilton T1	PCV+, (S)CMV+	Blower	Hamilton flow and pressure sensor	Active by blower	Low- and high-pressure	Coaxial tube	Flow

The O_2_ control system is either feed-forward defined by the low-pressure O_2_ supply setting (flow rate) or controlled in a closed loop with a high-pressure supply.

PEEP, positive end-expiratory pressure; PC-CMV, pressure-controlled continuous mandatory ventilation; VC-CMV, volume-controlled continuous mandatory ventilation; PCV+, pressure-controlled ventilation; (S)CMV+, synchronized controlled mandatory ventilation.

### Test environment

**Figure 1:**
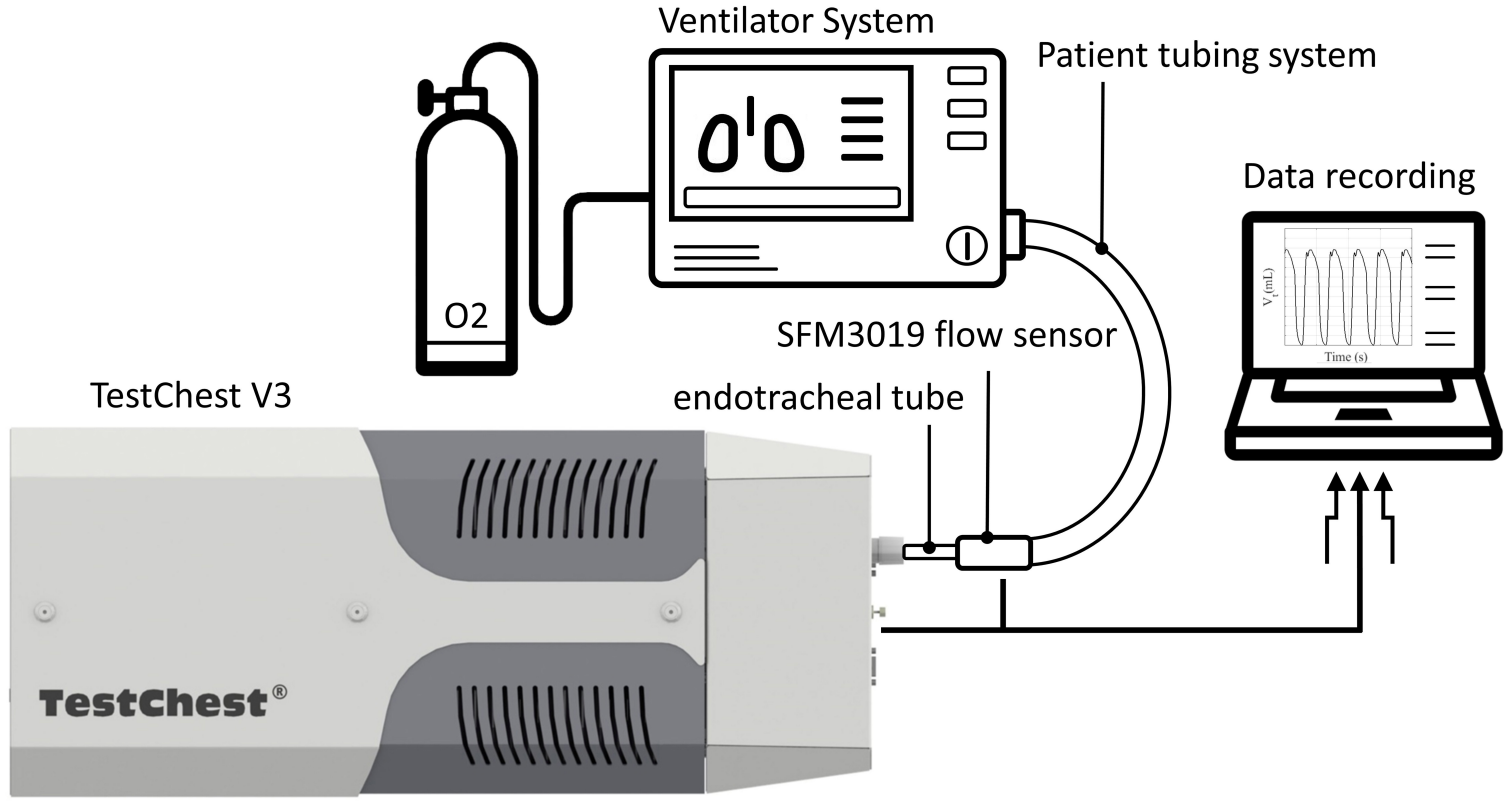
Schematic overview of the test environment for the experiments. Each ventilator system was attached via a tubing system and an endotracheal tube to the TestChest. The TestChest includes a pressure and an O_2_ sensor. In addition, a flow sensor SFM3019 was attached to the inlet of the TestChest to measure the actual tidal volume. A medical-grade O_2_ bottle (four bar) was used for the test of the O_2_ dynamics.

Each test described in this study was performed on a mechanical lung simulator (TestChest V3, Organis GmbH, Landquart, Switzerland). In brief, the TestChest can model different lung mechanics by varying airway resistance (R_aw_) and respiratory system compliance (C_rs_). Spontaneous breathing cycles can be set by means of a negative occlusion pressure (P_0.1_) and the respiratory rate (RR). The TestChest includes a pressure sensor measuring the airway pressure (P_aw_) and an O_2_ sensor with a response time <6 s for 90% of the final value placed in the airway. A flow sensor SFM3019 (Sensirion AG, Stäfa, Switzerland) was placed at the inlet of the TestChest to measure the resultant air flow. The ventilators were connected to the TestChest *via* an endotracheal tube (ETT) (SafetyClear I.D. 7.5, Teleflex Medical GmbH, Fellbach, Germany), as shown in Figure 1. For the efficiency of the O_2_ use and the response to a change of O_2_ supply, a medical-grade O_2_ tank was used, which includes an adjustable flow rate valve for low-pressure supply and a high-pressure port (four bar).

### Test protocol and experiments

The test protocol is divided into three categories: testing of (1) the accuracy of control systems and instruments, (2) the use of O_2_ and the response to a change in O_2_ supply, and (3) the ventilator triggering. The TestChest was calibrated with its internal program prior to the initiation of the study.

#### Accuracy of the control systems and instruments

The tests of the accuracy of the control systems and instruments assess the settings of the ventilators in the pressure- and volume-controlled modes. The TestChest was set to apnea, and the patient trigger signals of each ventilator were either switched off or set to the highest possible value. Each ventilator was tested at three different peak inspiratory pressure (PIP) or tidal volume (V_t_) settings with successively varying parameters to identify potential discontinuities and correlations. In total, 33 different settings were tested for the accuracy of the control systems and instruments. The base settings of the mechanical lung simulate a standard male adult (body height of 175 cm and weight of 68 kg) (15). The detailed settings are listed in Table 2. All measurements were recorded at steady state for 60 s.

**Table 2:** Ventilator settings for the individual tests performed.

**Ventilator settings**					**TestChest parameters**	
PIP*(cmH_**2**_O)	Vt**(mL)	**RR (bpm)**	**PEEP (cmH_**2**_O)**	**I:E ratio**	**C_**rs**_ (mL cmH_**2**_O^**−1**^)**	**R_**aw**_ (cmH_**2**_O s L^**−1**^)**
20 | 25 | 30	350 | 400 | 450	**10** | 15 | 20	5 |**10** | 15	1:1 |**1:2** |1:3	15 | 30 | **54**	**5** | 20 | 50
20 | 25 | 30	350 | 400 | 450	10 | **15** | 20	5 |**10** | 15	1:1 |**1:2** |1:3	15 | 30 | **54**	**5** | 20 | 50
20 | 25 | 30	350 | 400 | 450	10 | 15 | **20**	5 |**10** | 15	1:1 |**1:2** |1:3	15 | 30 | **54**	**5** | 20 | 50
20 | 25 | 30	350 | 400 | 450	**10** | 15 | 20	**5** |10 | 15	1:1 |**1:2** |1:3	15 | 30 | **54**	**5** | 20 | 50
20 | 25 | 30	350 | 400 | 450	**10** | 15 | 20	5 |10 | **15**	1:1 |**1:2** |1:3	15 | 30 | **54**	**5** | 20 | 50
20 | 25 | 30	350 | 400 | 450	**10** | 15 | 20	5 |**10** | 15	**1:1** |1:2 |1:3	15 | 30 | **54**	**5** | 20 | 50
20 | 25 | 30	350 | 400 | 450	**10** | 15 | 20	5 |**10** | 15	1:1 |1:2 |**1:3**	15 | 30 | **54**	**5** | 20 | 50
20 | 25 | 30	350 | 400 | 450	**10** | 15 | 20	5 |**10** | 15	1:1 |**1:2** |1:3	15 | **30** | 54	**5** | 20 | 50
20 | 25 | 30	350 | 400 | 450	**10** | 15 | 20	5 |**10** | 15	1:1 |**1:2** |1:3	**15** | 30 | 54	**5** | 20 | 50
20 | 25 | 30	350 | 400 | 450	**10** | 15 | 20	5 |**10** | 15	1:1 |**1:2** |1:3	15 | 30 | **54**	5 | **20** | 50
20 | 25 | 30	350 | 400 | 450	**10** | 15 | 20	5 |**10** | 15	1:1 |**1:2** |1:3	15 | 30 | **54**	5 | 20 | **50**

The gray-shaded settings represent the parameters changed with regard to the base setting of the ventilator (RR = 10 bpm, PEEP = 10 cmH_2_O, I:E = 1:2) and the test lung setting (C_rs_ = 54 mL/cmH_2_O, R_aw_ = 5 cmH_2_O /(L/s)). Each test was performed with a PIP of 20 cmH_2_O, 25 cmH_2_O, and 30 cmH_2_O and a Vt of 350 mL, 400 mL, and 450 mL for the pressure-controlled (^*^) and volume-controlled modes (^**^), respectively. In total, 33 different settings were tested. The bold values in the table denote the set value of the parameter in the corresponding test scenario.

PIP, peak inspiratory pressure; Vt, tidal volume; PEEP, positive end expiratory pressure; RR, respiratory rate; I:E, inspiratory to expiratory ratio; Crs, respiratory compliance; Raw, airway resistance.

#### Efficiency of oxygen use and response to a change in oxygen supply

The O_2_ dynamic tests are divided depending on the intended use of the ventilator either into low- or high-pressure supply. The V_t_ of each ventilator was set to 450 mL (6–8 mL per kilogram of body weight) (16), or the PIP was adjusted accordingly for the pressure-controlled ventilators. The RR was set to 10 bpm, the PEEP to 10 cmH_2_O, and the I:E to 1:2. For the low-pressure tests, the O_2_ supply flow was set to 2 L min^−1^, 4 L min^−1^, and 6 L min^−1^, respectively, and for the high-pressure tests, the desired O_2_ concentration was set in the user interface of the ventilator (40, 60, 80, and 100%). The resulting O_2_ concentration was measured *via* the integrated O_2_ sensor of the TestChest at steady state.

In addition, the t_90_ values of each ventilator were tested. It is defined as the time required for the O_2_ supply to reach 90% of the set O_2_ concentration measured by the O_2_ sensor in the TestChest. The TestChest was initially ventilated with ambient air. Then, the O_2_ supply was either set to a flow rate of 12 L min^−1^ (low pressure) or to an O_2_ concentration of 100% (high pressure) in the ventilator UI.

#### Ventilator trigger signals

Spontaneous breathing efforts were simulated by the TestChest by generating negative inspiratory pressures of P_0.1_ = 2 cmH_2_O/100 ms and P_0.1_ = 4 cmH_2_O/100 ms, respectively, at a respiratory rate of 10 bpm. Ventilators were set to a PEEP of 10 cmH_2_O, an I:E of 1:2, an RR of 8 bpm, and a PIP of 20 cmH_2_O for the pressure-controlled ventilators and 450 mL for the volume-controlled ventilator. The trigger signal was set to the lowest sensitivity threshold without causing auto triggering (17–20). Flow trigger sensitivity for the Hamilton T1 was set at 1 L min^−1^, for the HEV at 0.5 L min^−1^, and for the breathe at 1 L min^−1^. For the GirVent, a pressure trigger sensitivity of 0.1 cmH_2_O was set.

**Figure 2:**
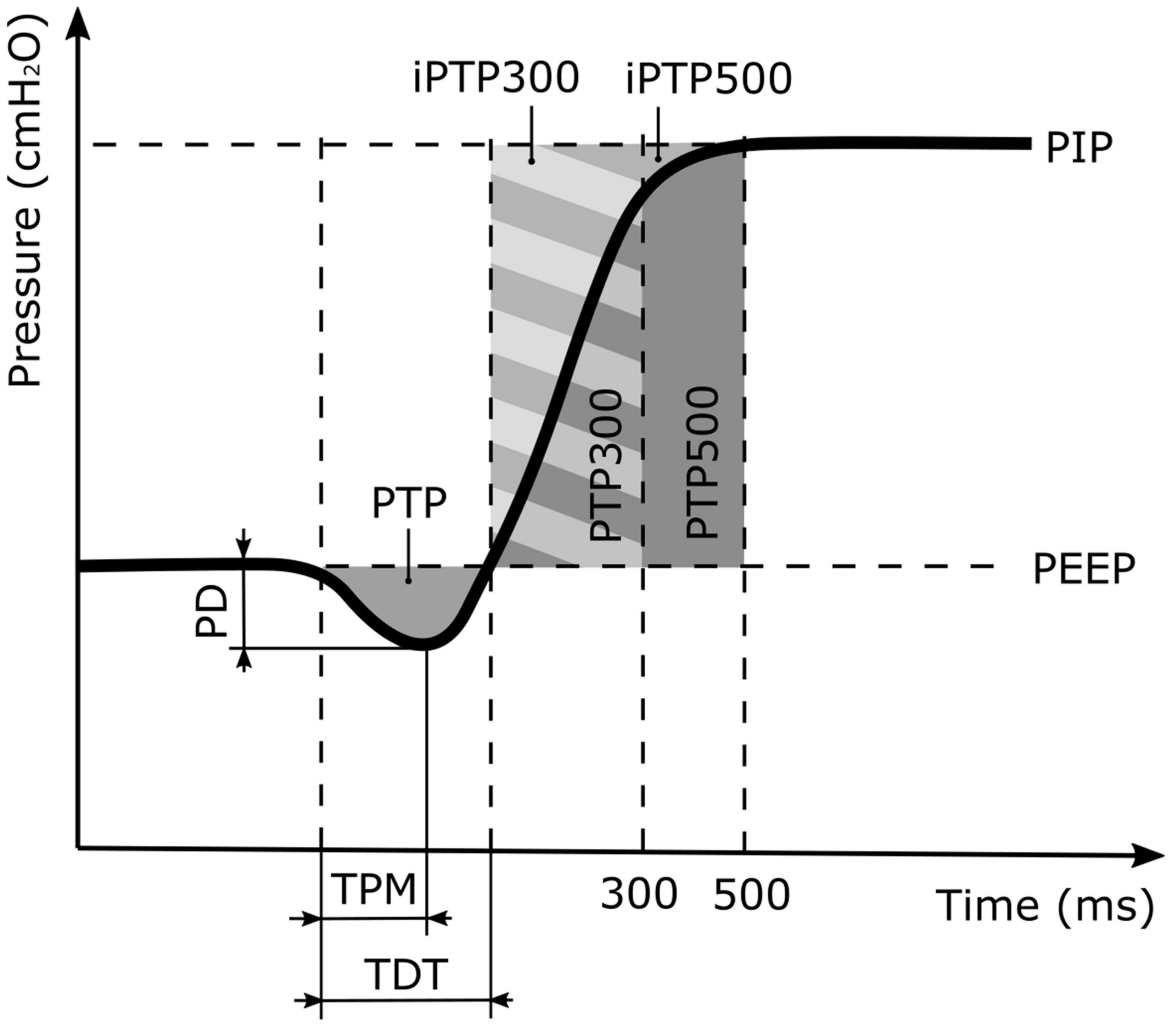
Illustration of the trigger response and the pressurization capacity parameters, where PD is the pressure drop of the patient effort. TPM is the time between the start of the patient inspiratory to the pressure minimum and TDT the time when the inspiratory pressure equals the positive end expiratory pressure (PEEP) value. PTP is the pressure time capacity defined as the area enclosed by the PD and the TDT. It describes the inspiratory patient effort prior to the ventilator support. The PTP300 and PTP500 values denote the pressurization capacity of the ventilator for the first 300 ms and 500 ms of the ventilation support cycle, respectively. The corresponding ideal areas of the pressurization capacity are denoted as iPTP300 and iPTP500.

Triggering performance is characterized by four defined parameters, as shown in Figure 2: (1) The maximum pressure drop (PD) from the PEEP indicates the inspiratory effort required to trigger ventilation. (2) The time from the beginning of the patient breath to the pressure minimum (TPM) represents the response time of the ventilator. (3) The trigger delay time (TDT) is the time required from the beginning of the patient breath until the P_aw_ recovers to the PEEP value. (4) The pressure time product (PTP) is the integral of the pressure curve during the TDT (17–20). The pressurization capacity of a ventilator is described by the ratio of the ideal PTP (iPTP) to the actual PTP during the first 300 ms (PTP300) or 500 ms (PTP500) after the P_aw_ exceeds the PEEP level. The ratios are denoted as PC300 and PC500, respectively.

### Signal processing and statistics

Signals were recorded *via* the TestChest V3 and the SFM3019 mass flow sensor at 100 Hz and processed in MATLAB R2020a (The Mathworks Inc., Natick, USA). The data of each experiment were shifted and windowed to a length of 50 s to eliminate phase shift differences between the single measurement sets, as shown in Table 2. The signal of P_aw_ and the flow measured in the TestChest (Q_TestChest_) were processed using a Gaussian filter with, a window length of 200 ms (20 samples). The RR was calculated using the time difference between two onsets of the inspiratory flow. The onset of the inspiratory flow starts when Q_TestChest_ reached a specified threshold. This threshold was defined per ventilator as the most sensitive value at which the fluctuation of Q_TestChest_ does not cause an onset detection. The inspiratory time (TI) and expiratory time (TE) are defined as the time during which Q_TestChest_ is above the positive threshold (inspiratory flow) and below the negative threshold (expiratory flow), respectively.

For each test performed, P_aw_ was measured inside the TestChest (Figure 1); hence, the PIP and PEEP displayed in the results report the pressure recorded downstream to the ETT. The PIP was detected as the peak value of P_aw_ averaged over 40 ms (four samples) symmetrically around the detected point signal. The PEEP value was determined as the minimum value of P_aw_ in the time interval of two consecutive breaths that was closer to the inspiration of the latter breath. The V_t_ was calculated by integrating the flow sensor signal. I:E is defined as the ratio of inspiratory time (T_I_) to expiratory time (T_E_) (T_I_:T_E_).

A Gaussian filter with a window length of 30 s (3,000 samples) was applied to the FiO_2_ signal recorded by the TestChest. The filtered signal averages the FiO_2_ values, while the unfiltered signal shows the FiO_2_ fluctuations during each breath cycle. The starting time of the t_90_ test was set when the unfiltered FiO_2_ signal exceeded an FiO_2_ of 22%. Maximum O_2_ step response and the 90% O_2_ step response were determined using the filtered signal.

Parameters for the trigger signals (PD, TPM, TDT, PTP) were evaluated from the P_aw_ signal. The start of inspiration is defined when exceeding 10% of the maximum gradient recorded during the pressure drop in the inspiratory phase.

## Results

### Accuracy of the control systems and instruments

**Figure 3:**
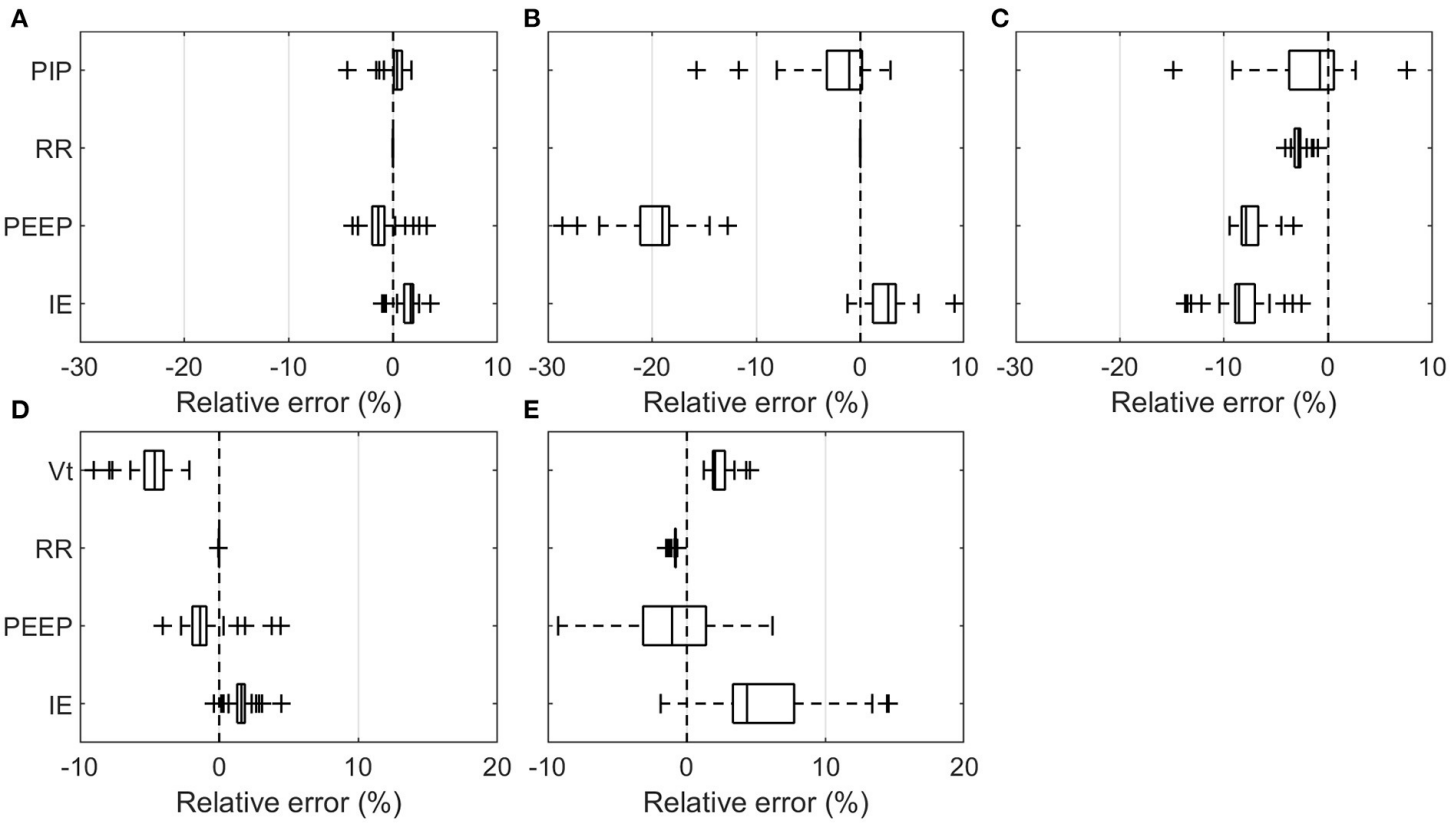
Boxplot representation of the relative error over all means of the individual measurement sets listed in Table 3. PIP, V_t_, RR, PEEP, and I:E are shown at varying RR, PEEP, I:E, C_rs_, and R_aw_ for the pressure- and volume-controlled ventilators. **(A)** Hamilton T1 (PCV+), **(B)** HEV, **(C)** GirVent, **(D)** Hamilton T1 ((S)CMV+), and **(E)** breathe. Each point represents the mean value of one experiment.

The overall relative errors of individual parameters recorded over all experiments (Table 2) are depicted in Figure 3. The relative error was calculated as the ratio of the measured error to the set value with the measured error being the difference between the value measured and the set value on the ventilator. Table 3 lists all the measured mean values, including standard deviation (SD). The PDVs showed generally larger relative mean errors and SD than the Hamilton T1 (PCV+ mode). In addition, more pronounced outliers were observed at individual measurement settings. Only the V_t_ measured in the breathe and the RR in all PDVs were in a range similar to those of the Hamilton T1, with no parameter changes having a substantial effect on the accuracy. The two passive PEEP valves used in the HEV and the breathe showed a larger variation than the actively applied PEEP in the GirVent.

**Table 3:** Mean and standard deviation (SD) of the relative error over all experiments and respiratory cycles for RR, PEEP, I:E, and PIP or Vt for the pressure-controlled and volume-controlled ventilators, respectively.

	**Pressure-controlled mode**			**Volume-controlled mode**	
**Ventilator /parameter**	**Hamilton T1 (PCV+)**	**GirVent**	**HEV CERN**	**Hamilton T1 ((S)CMV+)**	**breathe**
	**Mean ±SD (%)**	**Mean ±SD (%)**	**Mean ±SD (%)**	**Mean ±SD (%)**	**Mean ±SD (%)**
PIP (cmH_2_O)	0.26, 1.12	−1.89, 4.08	−2.19, 3.94	–	–
V_t_ (mL)	–	–	–	−4.72, 1.63	2.27, 0.89
RR (bpm)	0.02, 0.08	−2.84, 1.26	0.01, 0.09	−0.02, 0.17	−0.89, 0.32
PEEP (cmH_2_O)	−1.15, 1.16	−6.34, 1.33	−19.51, 3.04	−0.25, 1.44	−0.73, 4.00
I:E (-)	1.42, 1.05	−8.28, 2.91	2.55, 1.91	1.64, 0.93	5.76, 4.20

PCV+, pressure-controlled ventilation; (S)CMV+, synchronized controlled mandatory ventilation.

**Figure 4:**
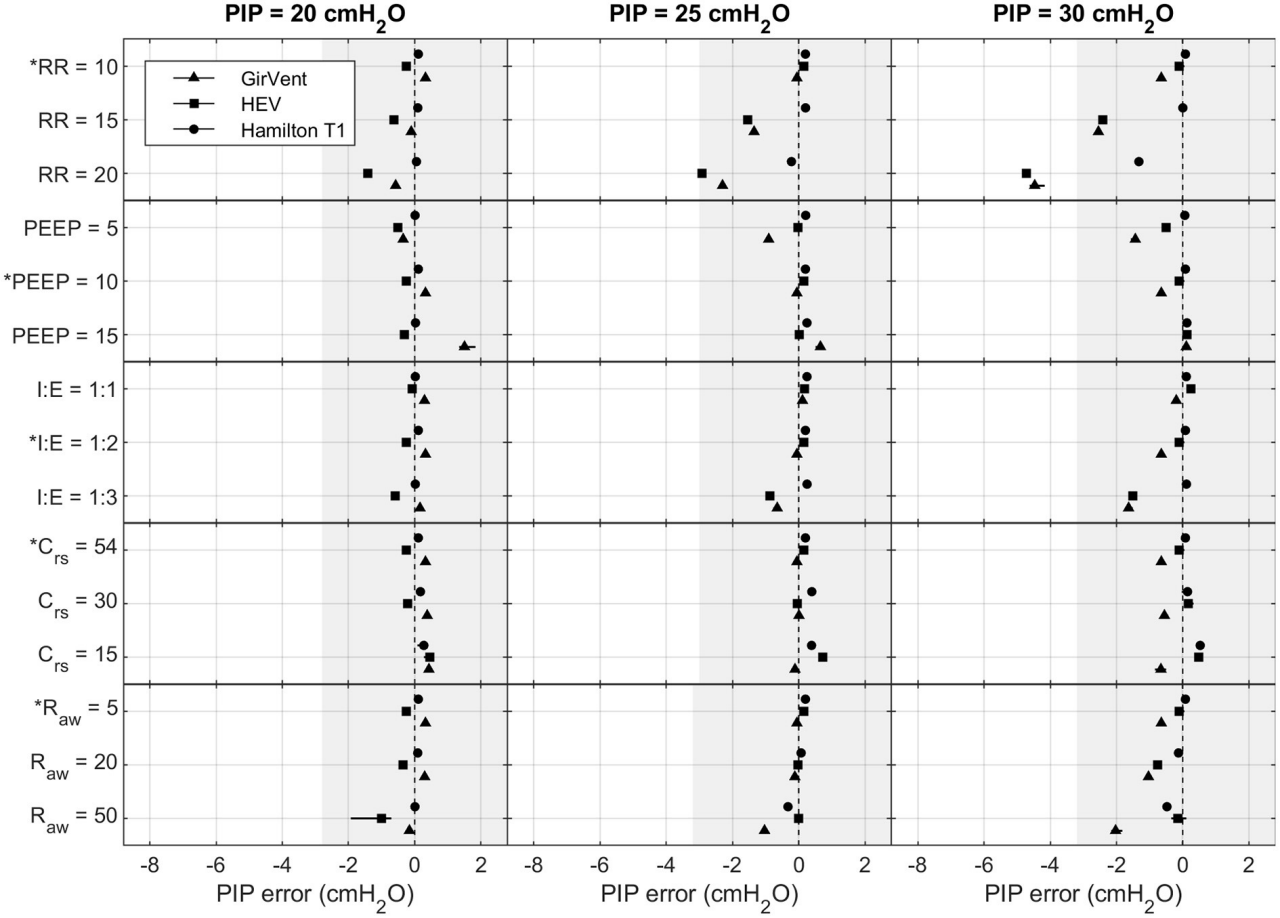
Measured PIP error in the Hamilton T1 (PCV+ mode), the HEV, and the GirVent: effects of altering PIP (cmH_2_O), RR (bpm), PEEP (cmH_2_O), I:E (ratio), Crs (mL cmH_2_O^−1^), and Raw (cmH_2_O s L^−1^). The base setting is indicated by ^*^. The markers and lines denote the mean error and the maximum and minimum errors, respectively. The grayed-out area indicates the acceptable performance tolerance according to the ISO 80601-2-12 norm and the MHRA specification.

**Figure 5:**
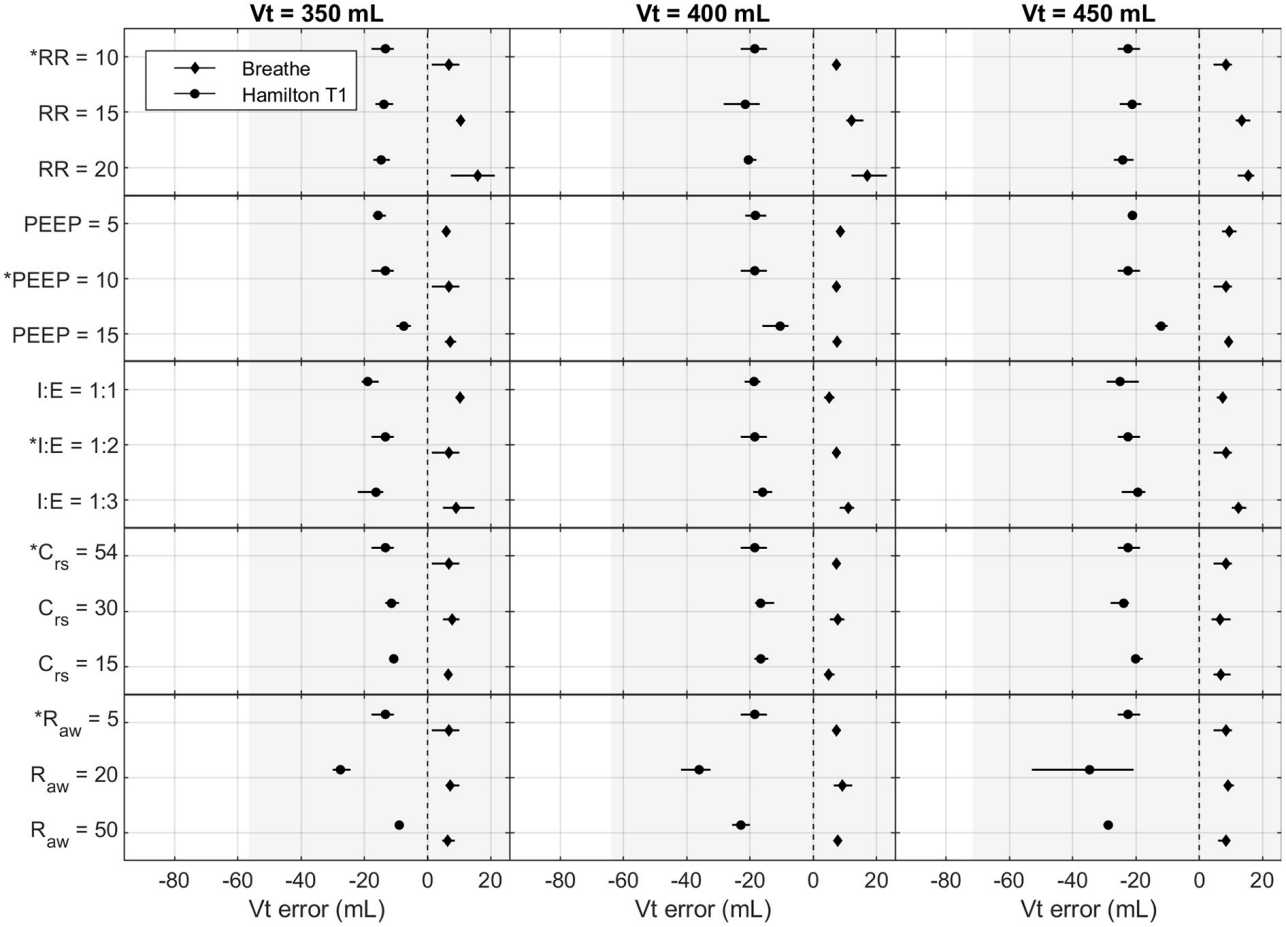
Measured Vt error in the Hamilton T1 ((S)CMV+ mode) and the breathe: effects of altering Vt (mL), RR (bpm), PEEP (cmH_2_O), I:E (ratio), Crs (mL cmH_2_O^−1^), and Raw (cmH_2_O s L^−1^). The base setting is indicated by ^*^. The markers and lines denote the mean error and the maximum and minimum errors, respectively. The grayed-out area indicates the acceptable performance tolerance according to the ISO 80601-2-12 norm and the MHRA specification.

Figures 4, 5 show the effects of the varying parameters on the PIP and the V_t_. An increase in the RR, a decrease in the inspiratory time, and an increase in R_aw_ showed the largest influence on the accuracy of the PIP. Moreover, the largest PIP error in each pressure-controlled ventilator was observed in the same setting of a PIP of 30 cmH_2_O and an RR of 20 bpm. By contrast, in the volume-controlled mode, only increasing the RR (breathe) or increasing R_aw_ (Hamilton T1 (S)CMV+ mode) had a prominent influence on the V_t_ error. Other parameter changes had only a marginal impact on the accuracy of the parameters tested. Detailed plots and data of each measurement and parameter change, as well as the flow profiles, recorded for the base setting for each ventilator are provided in the Supplementary material.

The ISO 80601-2-12 norm and the MHRA specify an acceptable performance tolerance of ± *(2* + *(4 % of the actual reading)) cmH*_2_*O* for the pressure value and ± *(4,0* + *(15% of the actual volume expired through the patient connection port)) mL*. The acceptable tolerance is given in Figures 4, 5. For the respiratory rate and I:E, no performance tolerance was stated in the norm. Therefore, for the tests performed in this study, we defined a tolerance of 5 and 10%, respectively.

### Oxygen dynamics

**Figure 6:**
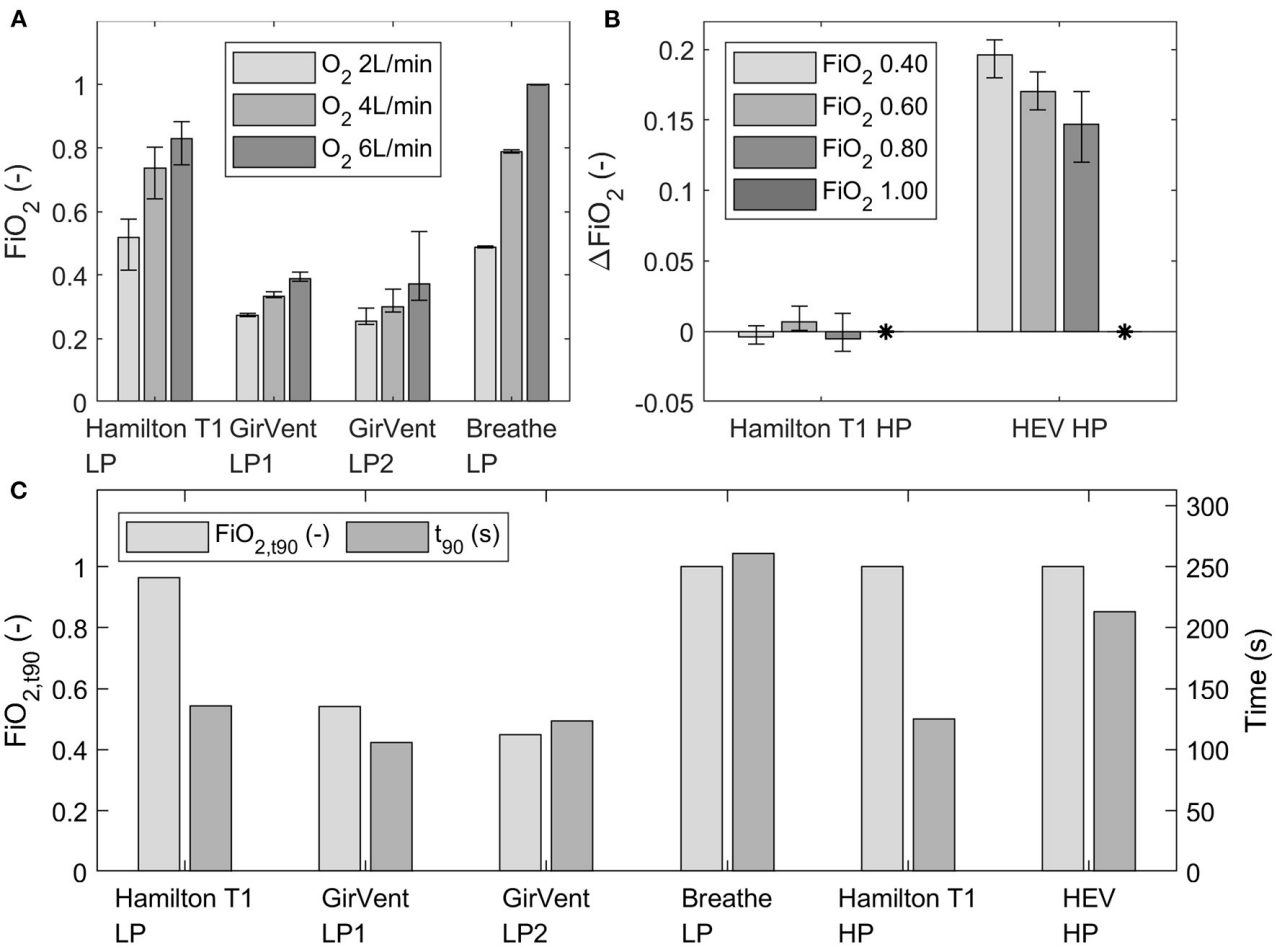
**(A)** Mean FiO_2_ at three O_2_ flow rates (2 L min^−1^, 4 L min^−1^, and 6 L min^−1^) for the ventilators with low-pressure O_2_ supply, depicted over the last 30 s of steady state. **(B)** Measured ΔFiO_2_ in the Hamilton T1 and HEV (high-pressure port) to the set FiO_2_ concentrations in the UI (0.40, 0.60, 0.80 and 1.00). The measured ΔFiO_2_ with an FiO_2_ setting of 1.00 in the UI is indicated by ^*^. The error bars indicate the fluctuation (minimum and maximum) observed at steady state. **(C)** Achieved FiO_2_, and t_90_ values at an O_2_ flow rate of 12 L/min (low pressure) or at a set O_2_ concentration of 100% (high pressure) of each ventilator.

The achieved fractions of inspired O_2_ (FiO_2_) at three O_2_ flow rates (2 L min^−1^, 4 L min^−1^, and 6 L min^−1^) for the low- and high-pressure supply (four bar) of each ventilator are presented in Figure 6. Fluctuations of FiO_2_ were observed in each ventilator and are indicated by error bars (minimum and maximum values). Generally, since the results are all relative values obtained by using the O_2_ sensor in steady state and the tests were all performed with the same O_2_ sensor, the delay of the sensor was neglectable.

The achieved mean FiO_2_ at steady state with the breathe was in a similar range or higher than that achieved with the Hamilton T1 with the low-pressure supply. By contrast, FiO_2_ measured with the GirVent was considerably low in the current design stage. In the tests with the high-pressure O_2_ supply, the Hamilton T1 achieved the intended O_2_ concentration with minor deviations from the set value. FiO_2_ provided by the HEV was constantly higher than the set concentration in the graphic UI.

The t_90_ values for the low-pressure supply (12 L min^−1^) and the high-pressure supply (4 bar, O_2_ at 100%), as well as the FiO_2_ achieved in the airway (FiO_2, t90_), are shown in Figure 6. GirVent showed for both O_2_ ports (LP-1 and LP-2) t_90_ values similar to those of the Hamilton T1; however, the achieved FiO_2, t90_ values were also lower than those of the other PDVs. For the breathe (LP O_2_ port) and the HEV (HP O_2_ port), the t_90_ values were higher than the values of the corresponding ports in the Hamilton T1, but both ventilators achieved FiO_2, t90_ of 1.00.

### Trigger signals

**Figure 7:**
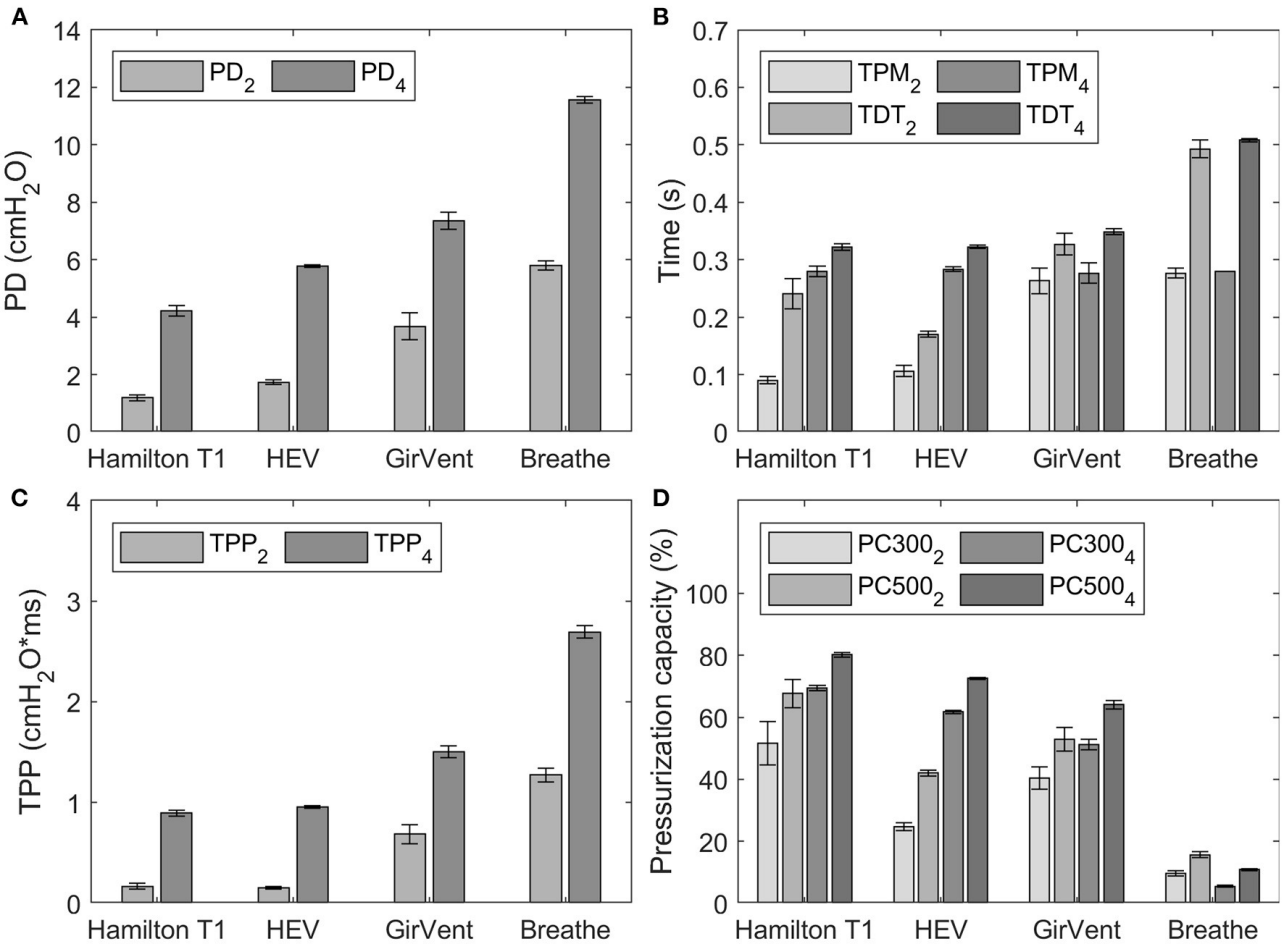
Results of the trigger characteristics for occlusion pressure values (P0.1) of 2 cmH_2_O/100 ms and 4 cmH_2_O/100 ms, indicated by the subscript in the corresponding legend. Error bars indicate the standard deviation. **(A)** pressure drop (PD), **(B)** time to pressure minimum (TPM) and trigger delay time (TDT), **(C)** time pressure product (TPP), and **(D)** pressurization capacity after 300 ms and 500 ms (PC300 and PC500).

The trigger signals and pressurization characteristics of each ventilator for the two P_0.1_ level set (2 cmH_2_O/100 ms and 4 cmH_2_O/100 ms) are shown in Figure 7. Exact values for all measurements are depicted in the Supplementary material.

For both values of P_0.1_, PD and TDT values observed in the PDVs were generally larger than those of the reference ventilator. The HEV presented the smallest difference to the reference ventilator and achieved faster TDT at a P_0.1_ of 2 cmH_2_O/100 ms.

The pressurization capacity of the PDVs was notably lower than that of the reference ventilator, which achieved values between 51.5 and 80.1%. The HEV presented values between 24.7% (PC300) and 72.5% (PC500), while those of the GirVent ranged between 40.4 and 64.0%, respectively. The values observed in the breathe ranged between 5.4 and 15.6%.

## Discussion

The test protocol presented was designed over several iterations in conjunction with the PDVs. It covers the most essential aspects for the first development phases of PDVs according to the MHRA specification. The chosen testing aspects—the accuracy of controls, the oxygen dynamics, and the trigger signals—are, from a technical point of view, the most crucial aspects in the first development phases. Additional features such as the basic alarms and monitoring are also of importance, although these can be addressed in the later stages of development.

The test protocol presented offers a compact and comprehensible evaluation of ventilators compared to the current guidelines and norms (5, 12). It serves as a complementary aid to the standard and covers the relevant aspects for the early development phases of ventilators. The automated plotting of the individual test scenarios allowed to see specific trends and provide conclusions for improvement. Various characteristics and the key differences of PDVs with different functional principles can be distinguished. The use of a reference ventilator allowed continuous comparison of the PDVs with a state-of-the-art ventilator. Overall, the most prominent differences and characteristics of the PDVs tested could be elaborated.

### Accuracy of the control systems and instruments

Generally, the design stage tested of the PDVs showed satisfactory accuracy of the control systems and instruments (12). Nonetheless, individual mechanisms of the PDVs resulted in some strong outliers and in increased inaccuracies at individual test settings compared to those of the reference ventilator.

All ventilators showed a decelerating flow profile, except the breathe which showed an ascending flow profile. Hence, a direct comparison between the ventilators is not desirable. However, the results presented evaluate their accuracy regarding their main control variables, independent of their different functional principles.

The applied tests resulted in a critical differentiation of functional performance of the evaluated PDVs, such as the passive PEEP valves (HEV and breathe) showed larger relative errors and variations compared to the actively controlled PEEP by blowers (Hamilton T1 and GirVent). The PEEP generated by a passive PEEP valve is more prone to errors and potentially less stable under clinical conditions of respiratory diseases.

**Figure 8:**
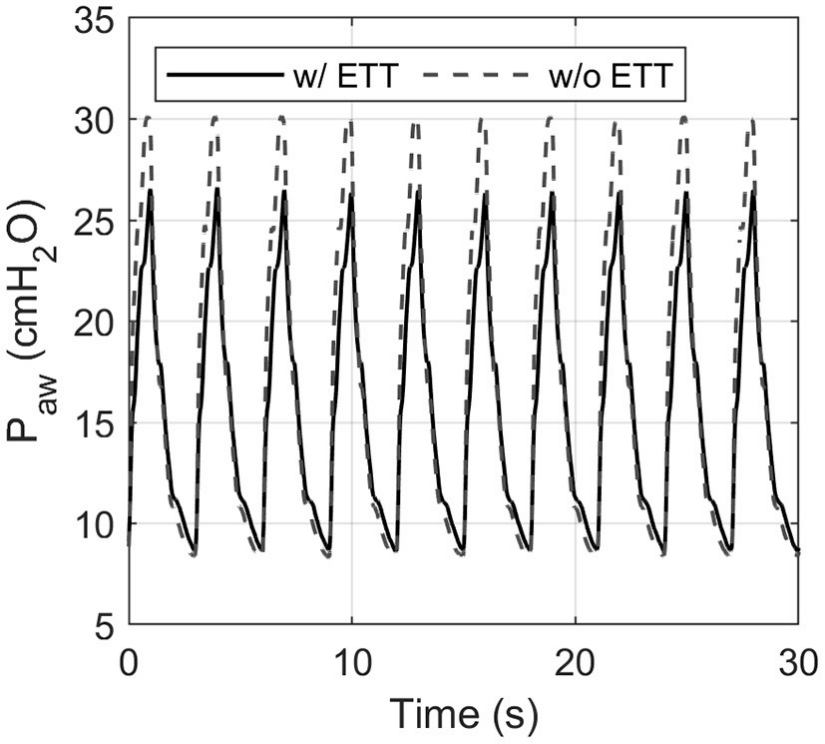
Influence of the endotracheal tube (ETT) on the measured airway pressure shown for the HEV ventilator. The ETT introduces additional flow resistance, resulting in a considerably lower peak pressure. This effect was evident in each ventilator tested.

Each pressure-controlled ventilator displayed maximum PIP error at a PIP of 30 cm H_2_O, with an RR of 20 bpm. As shown in Figure 4, the PIP error increases when inspiratory time is decreased. This effect is mainly caused by the resistance of the ETT used in the experiments. Figure 8 shows an example of the pressure profile (HEV ventilator) measured with and without an ETT in the setting PIP = 30 cm H_2_O and RR = 20 bpm.

### Oxygen dynamics

The main target regions for PDVs are environments with low or limited access to a supply of O_2_. The O_2_ performance with a limited flow feed-forward control is highly relevant for PDVs, and the economic use of O_2_ is of great importance. However, concerning clinical implications, it is crucial that a PDV is also capable of delivering a high O_2_ concentration.

According to the ISO 80601-2-12 norm and the MHRA specification, the accepted accuracy for the oxygen concentrations is ± 5% of the set value. As such, this can only be evaluated for the feedback control of the high-pressure O_2_ supply tests. However, the norm and the MHRA specification do not state any accepted performance for the low-pressure O_2_ efficiency.

In the low-pressure O_2_ supply mode (2, 4, 6 L min^−1^), the breathe achieved higher FiO_2_ at steady state than the Hamilton T1. The resuscitator bag serves as a large reservoir, which premixes air and O_2_, thereby allowing a homogeneous gas mix. However, this system has no other form of O_2_ control than the supply flow set. In sharp contrast, the GirVent achieved considerably lower FiO_2_ with both O_2_ port modes implemented. A solenoid valve is used here to limit the O_2_ injection exclusively to the inspiratory phase. This design results in a sparing use of O_2_; however, high FiO_2_ cannot be achieved. The HEV ventilator with the high-pressure O_2_ port showed a larger error than 5%, with continuous overshoot to the set FiO_2_. Thus, it requires additional tuning of the software and calibration of the O_2_ sensor. By contrast, the Hamilton T1 showed high accuracy in the high-pressure O_2_ tests, ranging within the 5% tolerance.

The t_90_ value represents the response to an FiO_2_ change. A limited increase in maximal FiO_2_ may result in a low t_90_ value, but it also indicates that the ventilator system does not manage to provide the patient with a high FiO_2_. For instance, a very low t_90_value was observed in the GirVent, but the maximal FiO_2_ achieved is likewise very low, whereas with the breathe and the HEV, a long t_90_ value was observed, but the achieved FiO_2_ concentrations approach 1.00. In general, the O_2_ operation modes of the HEV and the breathe show that premixing O_2_ in a reservoir is beneficial for a homogeneous O_2_ supply. In addition, the implementation of an O_2_ sensor is recommendable even if FiO_2_ can be regulated with the setting of the O_2_ source.

### Ventilator trigger signals

The correct detection of and fast reaction to a patient's breathing efforts is crucial to prevent patient–ventilator asynchrony, which may cause discomfort or even lung injury (21, 22). A higher initial flow after triggering results in a better pressurization capacity of the ventilator and in decreased patient effort (23, 24). Overall, the PDVs tested here detected the applied P_0.1_ levels and suitably triggered breathing cycles generated by the TestChest. Concerning the acceptable performance for the trigger detection and reaction, neither the ISO 80601-2-12 standard nor the MHRA specification specifies any tolerances.

Relative to results reported in commercial ventilators, our measurements showed generally higher TDT, including the Hamilton T1 (17–20). This may be explained by the additional resistance imposed by the ETT, which causes a restricted and delayed flow signal, which in turn requires more sensitive triggering. Compared to studies that used a PEEP level of 0 cmH_2_O or 5 cmH_2_O (17–20), the use of an increased PEEP level (10 cmH_2_O) requires a less sensitive trigger to avoid auto-triggering (17–20). This directly results in a slower detection and a longer TDT.

Although the HEV and the GirVent display slightly higher PD and TDT levels and lower pressurization capacities than the reference ventilator, the results are encouraging, also considering the fact that the devices are still in an early development state. Furthermore, Delgado et al. (18) reported pressurization capacities of different commercial ventilators in a similar magnitude to those we observed in the HEV and the GirVent.

### Strengths and limitations

In this study, we showed that the presented test protocol enables rapid and conclusive evaluations of PDVs during their early development phase. The test protocol and the evaluations of the individual parameters were compared with the performance of the Hamilton T1 as a reference system. The simplicity of the protocol in combination with the automated detection and evaluation algorithm, fast quantification of the performance, as well as iterative and test-oriented development of PDVs, is supported. Safety features required according to the norm ISO 80601-2-12:2020 (12) and the MHRA recommendation (5) were considered but are not comprehensively incorporated in the present study. The objective of this simplified protocol was to enable data-driven development based on realistic *in vitro* tests. However, our test protocol does not replace the tests required for certification.

The norm ISO 80601-2-12:2020 (12) states to use a linear resistance that accounts for the resistance induced by the ETT and the pulmonary system. However, the restricting cross-sectional area of an ETT introduces a relevant parabolic resistance and flow disturbance that might not be accounted for solely with a linear resistance implemented in the mechanical lung, especially at high flow rates. The impact of the ETT is also evident in the trigger signal evaluation. Overall, the use of ETTs is of critical importance as this represents clinical reality of the gold standard therapeutic strategy, and it is beneficial to investigate representative clinical scenarios.

Other limitations of the test protocol presented include the lack of testing of the efficiency of O_2_ use of each ventilator. To determine their actual efficiency, the amount of O_2_ used from the source to achieve the desired FiO_2_ needs to be measured. Also, the robustness of software and hardware of each ventilator ought to be assessed under high load. Last, the patient cycling that describes the transition from inspiration to expiration during assisted ventilation (25) was not measured in this study.

In comparison to *in vivo* testing, our test approach provides simplicity and complete experimental control. Nonetheless, it is physiologically limited, whereas *in vivo* tests provide more specific and reliable observation of biological effects but with strict regulations and compliance standards.

In conclusion, we presented a systematic test approach, which helps develop pandemic ventilators in a short time, independent of their ventilation principle. The test protocol covers the essential functional aspects for the initial development phase of PDVs. In addition, the automated and modular evaluation scripts allows for performing iterative investigations and drawing conclusions regarding specific ventilator characteristics.

## Data availability statement

The raw data of the Hamilton T1 supporting the conclusions of this article will be made available by the authors, without undue reservation.

## Ethics statement

Ethical review and approval was not required for this study in accordance with the local legislation and institutional requirements.

## Author contributions

The experiments were conceived and designed by NT and NSte. Data collection and analysis was performed by NT, NSte, and MZ. Literature search and study discussions were conducted by NT, NSte, MZ, MS, NSta, TE, and JH. The manuscript was prepared by NT and reviewed by NSte, MZ, NSta, MM, TE, JH, and MS. All authors contributed to the article and approved the submitted version.

## Funding

This project was financed by ETH Zürich and the Botnar Research Center for Child Health (COVent project). The sponsor had no role in the design or conduct of this research. Open access funding provided by ETH Zürich.

## Conflict of interest

Authors NT, NSte, MZ, MM, and MS with the Product Development Group Zurich, ETH Zürich, declare that they have been involved in the development of each PDV presented in this study by actively testing several iterations and providing the teams with feedback. No funding has been received by any of the teams associated to the PDVs. The remaining authors declare that the research was conducted in the absence of any commercial or financial relationships that could be construed as a potential conflict of interest.

## Publisher's note

All claims expressed in this article are solely those of the authors and do not necessarily represent those of their affiliated organizations, or those of the publisher, the editors and the reviewers. Any product that may be evaluated in this article, or claim that may be made by its manufacturer, is not guaranteed or endorsed by the publisher.

## Abbreviations

PDV: pandemic ventilators
PC-CMV: pressure-controlled continuous mandatory ventilation
VC-CMV: volume-controlled continuous mandatory ventilation
MHRA: Medicines and Healthcare Products Regulatory Agency
ETT: endotracheal tube
PEEP: positive end-expiratory pressure
PIP: peak inspiratory pressure
RR: respiratory rate
V_t_: tidal volume
R_aw_: airway resistance
C_rs_: respiratory compliance
P_aw_: airway pressure
Q_TestChest_: airflow into TestChest
T_I_: inspiratory time
T_E_: expiratory time
I:E: inspiratory to expiratory time ratio
FiO_2_: fraction of inspired O_2_
HP: high pressure
LP: low pressure
PD: pressure drop
TPM: time to pressure minimum
TDT: trigger delay time
PTP: pressure time product
PC: pressurization capacity.
