# Supplementary material for: Testing of pandemic ventilators under early and agile development
Source: Front Med Technol. 2022 Aug 16;4:899328. doi: 10.3389/fmedt.2022.899328 (PMC9424737; doi:10.3389/fmedt.2022.899328)
Supplement: Supplementary file 1 [file Table_1.pdf]

Supplementary Material

Testing of pandemic ventilators under early and agile development

1 GirVent Description

1.1 Design

The GirVent ventilator (GirTec AG, Sulgen, Switzerland) is a compact mechanical emergency ventilator (<5kg) intended for use in patients with acute or chronic respiratory failure or respiratory insufficiency (Figure 1). The pressure generated during inspiration as well as during expiration (PIP and actively generated PEEP) is provided by a turbine blower. There is a total of one port for the intake of fresh air and the exhaust of used air. The inspiratory and expiratory phases are controlled by the opening and closing of a solenoid valve inside the ventilator.

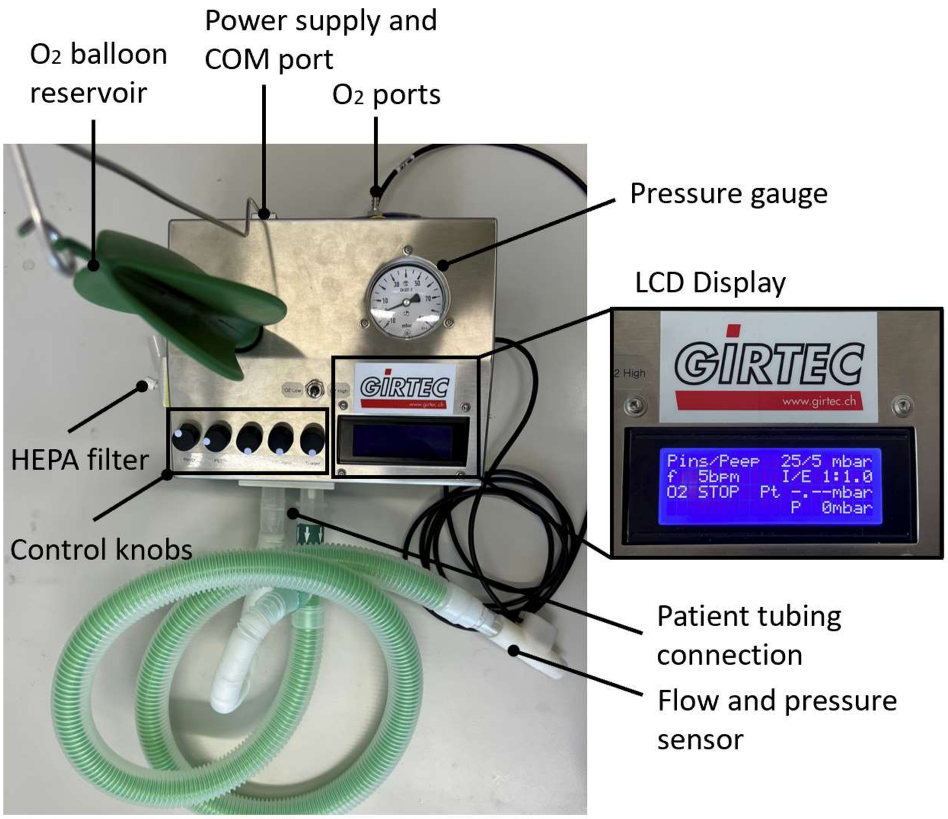

Supplementary Figure 1 Illustration of the GirVent ventilator. The ventilator has five control knobs for setting the parameters; plateau pressure, PEEP, respiratory rate, I:E ratio and the trigger sensitivity. The measured values with the flow and pressure sensors at the distal end of the patient tubing are displayed on an LCD screen. An additional pressure gauge indicates the pressure at the proximal end of the patient tubing.

The tested patient tubing system consists of a double lumen tube with a mass flow sensor and an integrated pressure sensor at the distal end of the tube. The tubing system is connected to two standard ports; for the intake of fresh air during inspiration and the exhaust of exhaled air.

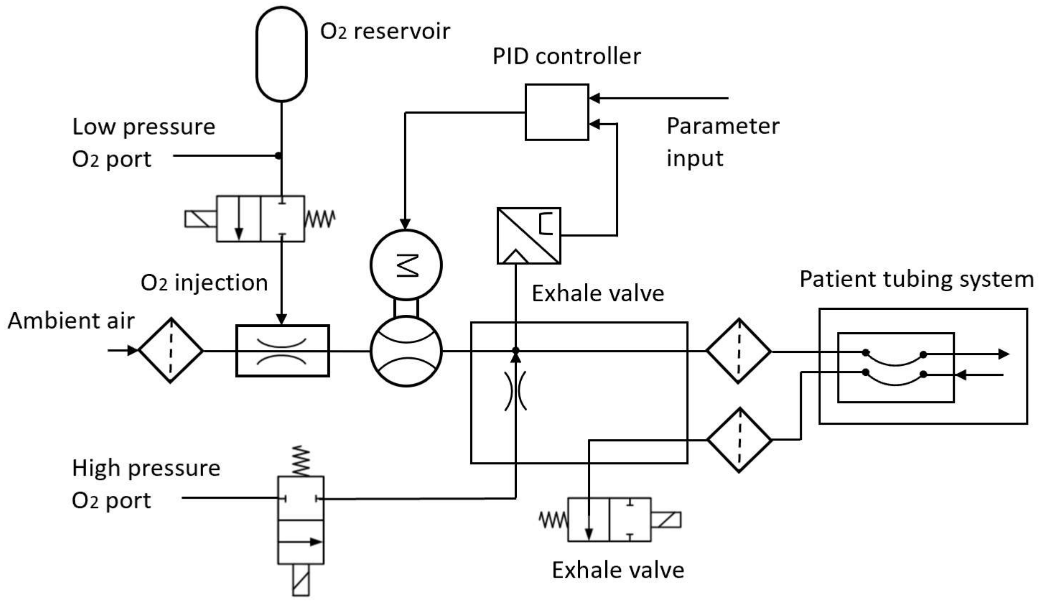

Supplementary Figure 2 Schematic representation of the operating concept of the girvent. The system can be operated either with low pressure O_2 or with high pressure O_2. The low pressure O_2 is injected into the patient circulatory system via a reservoir, while the high pressure O_2 is added via a throttle valve. A PID cotnroller regulates the applied pressure via the pressure sensor attached at the patient tubing system.

1.2 Controls and monitoring

Basic Controls

The parameters on the Grivent are set using analog rotary dials. Five parameters can be set on the ventilator; the plateau pressure can be set from 15-35 cmH_2O, the PEEP can be set from 2-15 cmH_2O, the respiratory rate can be set from 5-30 bpm, the I:E ratio can be set from 1:1-1:3, and the trigger sensitivity can be set from 0-2 mbar.

Pressure-Control mode and Trigger signals

At the time of the measurements for the study, the GirVent could be operated in one mode, the pressure-controlled continuous mandatory ventilation (PC-CMV). The pressure generated in the ventilation system (ventilator and patient tubing system) is controlled by an actively controlled solenoid valve. The expiration port of the valve is closed during inspiration and open during expiration. By controlling the opening and closing of the solenoid valve, the PEEP can be actively regulated. The pressure and flow sensor attached at the distal end of the patient tubing system is used for the internal feedback of the PID controller of the turbine blower. The pressure sensor is also used for triggering in assisted ventilation mode. The trigger signal is transmitted via an adjustable threshold through the measured negative pressure drop in the pressure sensor.

Oxygen supply

O_2 supply can be provided both from a pressure source (high-pressure mode) and admixture of O_2 from a low-pressure source via an injector (low-pressure mode). In the low-pressure mode, the O_2 source is connected to a standard connection for low-pressure oxygen (0.25 bar). The O_2 supplied with the low-pressure port is first distributed into a two-liter balloon reservoir and then aspirated during inspiration. The pressure in the balloon reservoir, hence, the amount of O2 supplied during inspiration, is controlled by the O_2 supply flow from the O_2 source. In high pressure mode, the pressure source is connected to a standard port for high pressure O_2 (max. 4.5 bar). The O_2 is injected into the patient tubing system during inspiration via a port on the inspiratory output of the ventilator.

Monitoring

The GirVent has a digital LCD display for monitoring all adjustable parameters. In addition, an analog manometer display continuously displays the instantaneous pump pressure. The digital display has four text lines; i.e. inspiratory pressure, PEEP, respiratory rate, I:E ratio, trigger pressure threshold, O_2 supply mode and for error messages or alarms. Further, the following alarms are implemented; inspiratory Paw exceeded or not achieved, end expiratory pressure exceeded or not achieved and failure of electricity or gas supply.

2 Differences to MHRA recommendation

Supplementary Table 1 Overview of the elements tested presented our test protocol and the MHRA specification. The MHRA specification covers a broad range of scenarios in the tests for accuracy of controls and systems. It covers 36 tests each for changing lung compliance, airway resistance, O_2 setting, tidal volume and PIP. However, the MHRA specification does not cover tests for trigger signals. Further requirements for PDVs are also covered in the MHRA specifications

	PDV test protocol	MHRA recommendation
	**Testing**	
Accuracy of controls and systems	33 tests (volume control)<br>33 tests (pressure control)	36 tests (volume control – compliance)<br>36 tests (volume control – resistance)<br>36 tests (volume control – tidal volume)<br>36 tests (pressure control – 15cmH_2O)<br>36 tests (pressure control – 30cmH_2O)
Trigger signals	Testing of pressure drop, time to pressure minimum, trigger delay time and pressure time product	not included
O_2 dynamics	Testing of t_{90}, high pressure O_2 accuracy and low pressure O_2 efficiency	O_2 accuracy test included in tests of accuracy of controls and systems
Pressure relief test	no	Tests confirmation that the pressure in the system does not exceed 80cmH_2O and alarm is activated
Closed suction test	no	Tests confirmation that the ventilator returns to default setting after vacuum is applied
	**Further requirements**	
General requirements	n.a.	yes
Gas and electricity	n.a.	yes
Infection control	n.a.	yes
Monitoring and alarms	n.a.	yes
Biological safety	n.a.	yes
EMC safety	n.a.	yes
Software safety	n.a.	yes

3 Accuracy of Controls and Instruments

3.1 Overview

Supplementary Table 2 Mean and standard deviation (SD) of the relative error over all experiments and respiratory cycles for RR, PEEP, I:E ratio and PIP or Vt for the pressure-controlled and volume-controlled ventilators, respectively.

Ventilator / Parameter	Pressure-controlled mode			Volume-controlled mode	
	Hamilton T1 (PCV+)	GirVent	HEV CERN	Hamilton T1 ((S)CMV+)	Breathe
	Mean ± SD (%)	Mean ± SD (%)	Mean ± SD (%)	Mean ± SD (%)	Mean ± SD (%)
PIP	0.26 ± 1.12	-1.89 ± 4.08	-2.19 ± 3.94	-	-
V_t	-	-	-	-4.72 ± 1.63	2.27 ± 0.89
RR	0.02 ± 0.08	-2.84 ± 1.26	0.01 ± 0.09	-0.02 ± 0.17	-0.89 ± 0.32
PEEP	-1.15 ± 1.16	-6.34 ± 1.33	-19.51 ± 3.04	-0.25 ± 1.44	-0.73 ± 4.00
I:E	1.42 ± 1.05	-8.28 ± 2.91	2.55 ± 1.91	1.64 ± 0.93	5.76 ± 4.20

3.2 Pressure-Controlled Ventilators

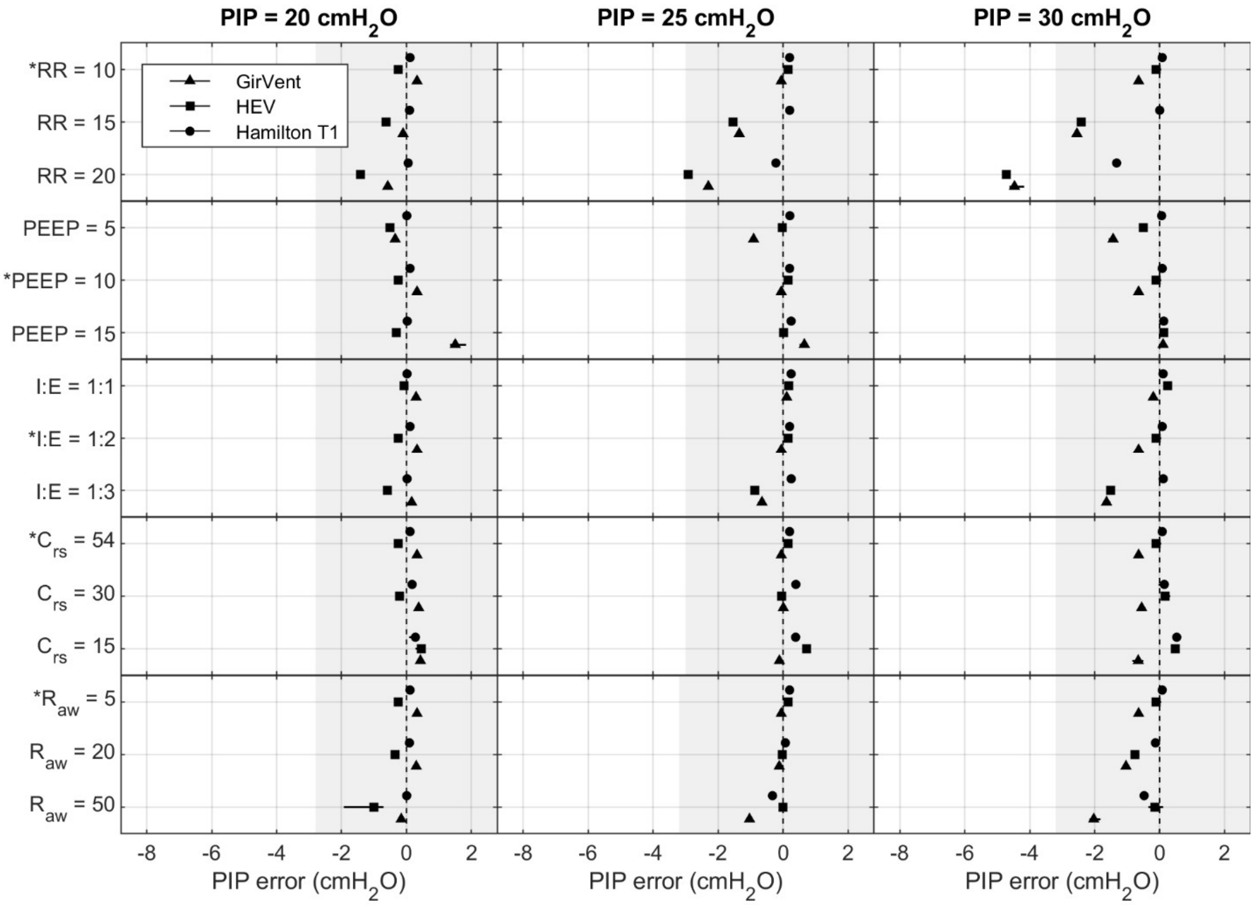

Supplementary Figure 3 Measured PIP error in the Hamilton T1 (PCV+ mode), the HEV and the GirVent: effects of altering PIP (cmH_2O), RR (bpm), PEEP (cmH_2O), I:E (ratio), Crs (mL/cmH_2O) and Raw ($cmH_2O/(L/s)$). Base setting indicated by *. The markers and lines denote the mean error and the maximum and minimum errors, respectively. The grayed-out area indicates the acceptable performance (ISO norm).

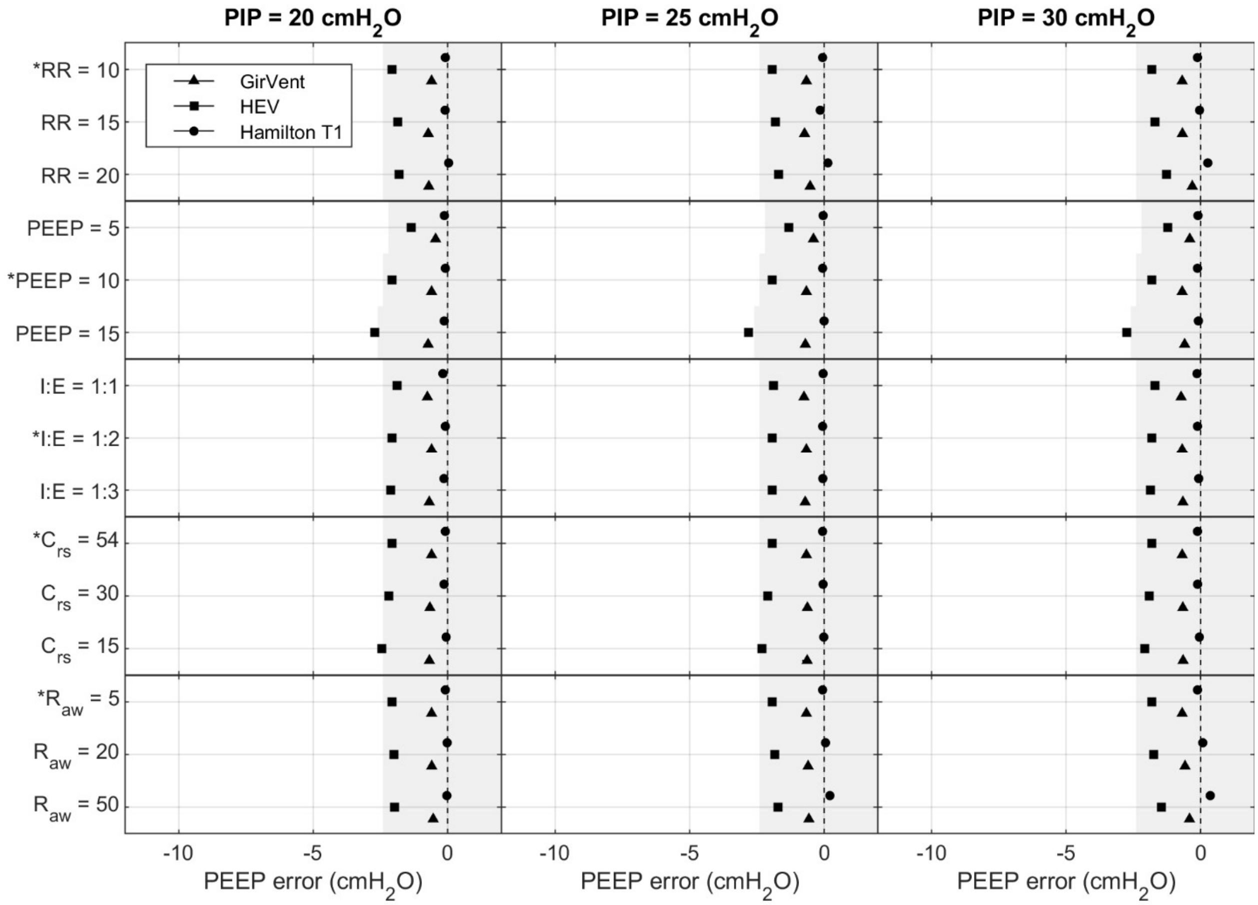

Supplementary Figure 4 Measured PEEP error in the Hamilton T1 (PCV+ mode), the HEV and the GirVent: effects of altering PIP (cmH_2O), RR (bpm), PEEP (cmH_2O), I:E (ratio), Crs (mL/cmH_2O) and Raw ($cmH_2O/(L/s)$). Base setting indicated by *. The markers and lines denote the mean error and the maximum and minimum errors, respectively. The grayed-out area indicates the acceptable performance (ISO norm).

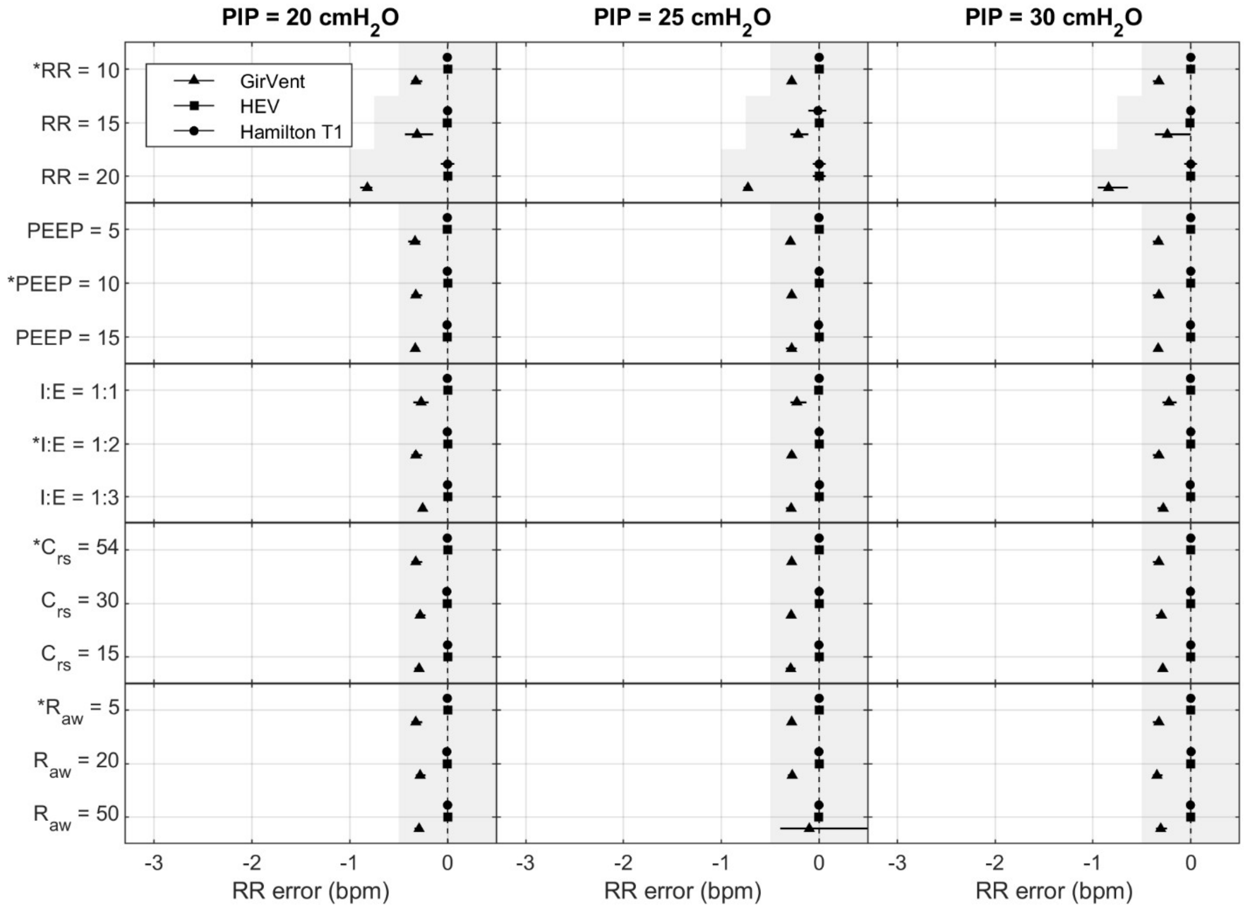

Supplementary Figure 5 Measured RR error in the Hamilton T1 (PCV+ mode), the HEV and the GirVent: effects of altering PIP (cmH_2O), RR (bpm), PEEP (cmH_2O), I:E (ratio), Crs (mL/cmH_2O) and Raw ($cmH_2O/(L/s)$). Base setting indicated by *. The markers and lines denote the mean error and the maximum and minimum errors, respectively. The grayed-out area defines the acceptable performance (±5%).

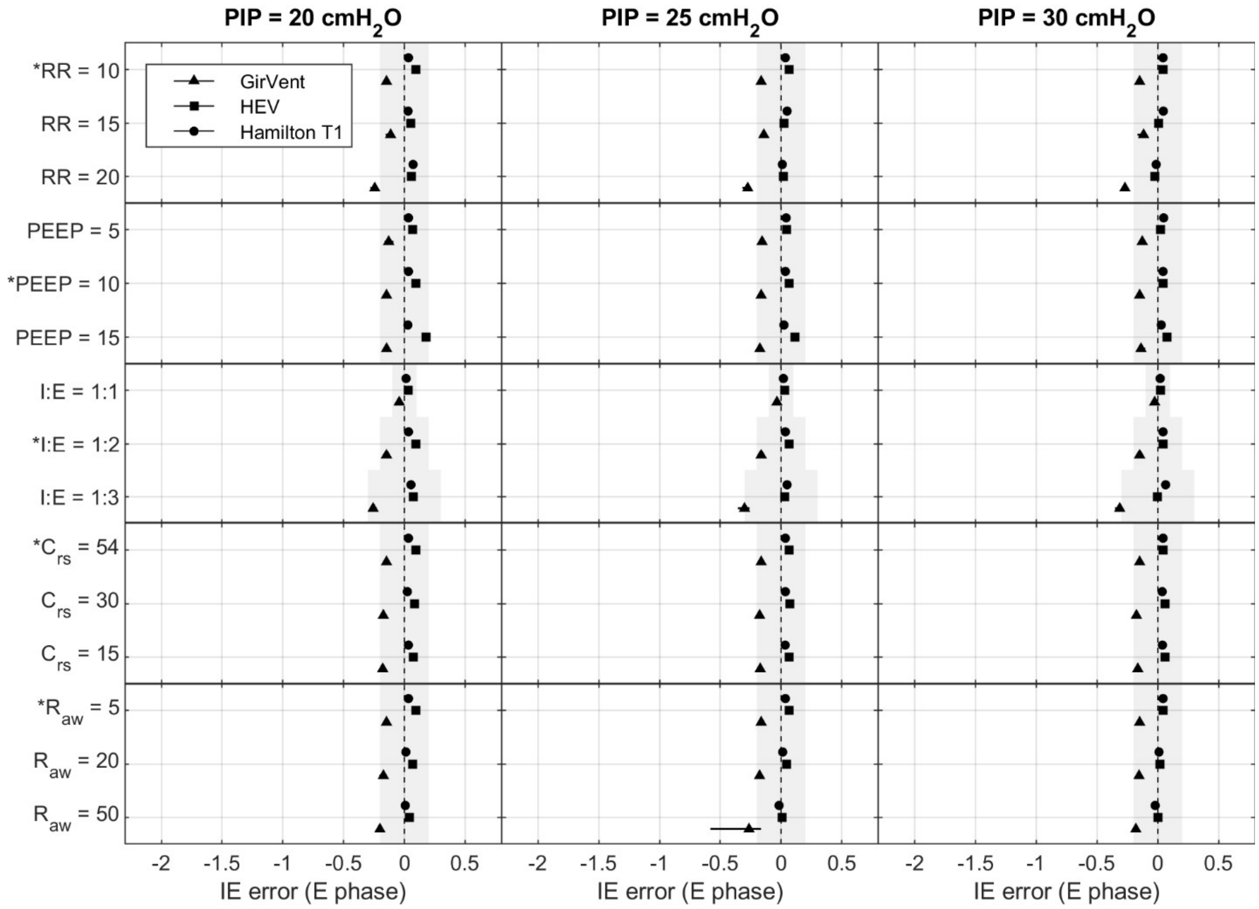

Supplementary Figure 6 Measured I:E error in the Hamilton T1 (PCV+ mode), the HEV and the GirVent: effects of altering PIP (cmH_2O), RR (bpm), PEEP (cmH_2O), I:E (ratio), Crs (mL/cmH_2O) and Raw ($cmH_2O/(L/s)$). Base setting indicated by *. The markers and lines denote the mean error and the maximum and minimum errors, respectively. The grayed-out area defines the acceptable performance (±10%).

3.3 Volume-Controlled Ventilators

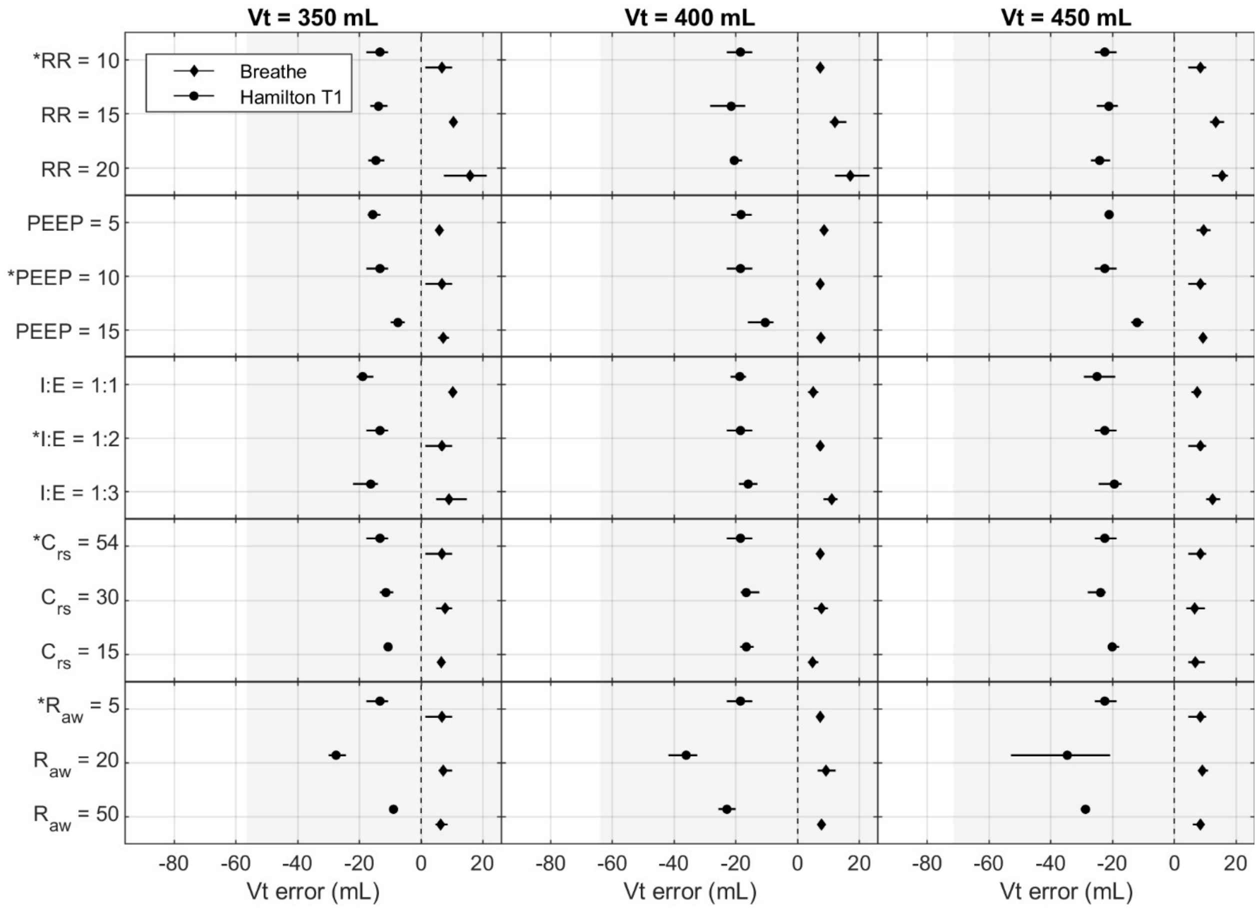

Supplementary Figure 7 Measured V_t error in the Hamilton T1 ((S)CMV+ mode) and the Breathe: effects of altering V_t (mL), RR (bpm), PEEP (cmH_2O), I:E (ratio), C_{rs} (mL/cmH_2O) and R_{aw} ($cmH_2O/(L/s)$). Base setting indicated by *. The markers and lines denote the mean error and the maximum and minimum errors, respectively. The grayed-out area defines the acceptable performance (ISO norm).

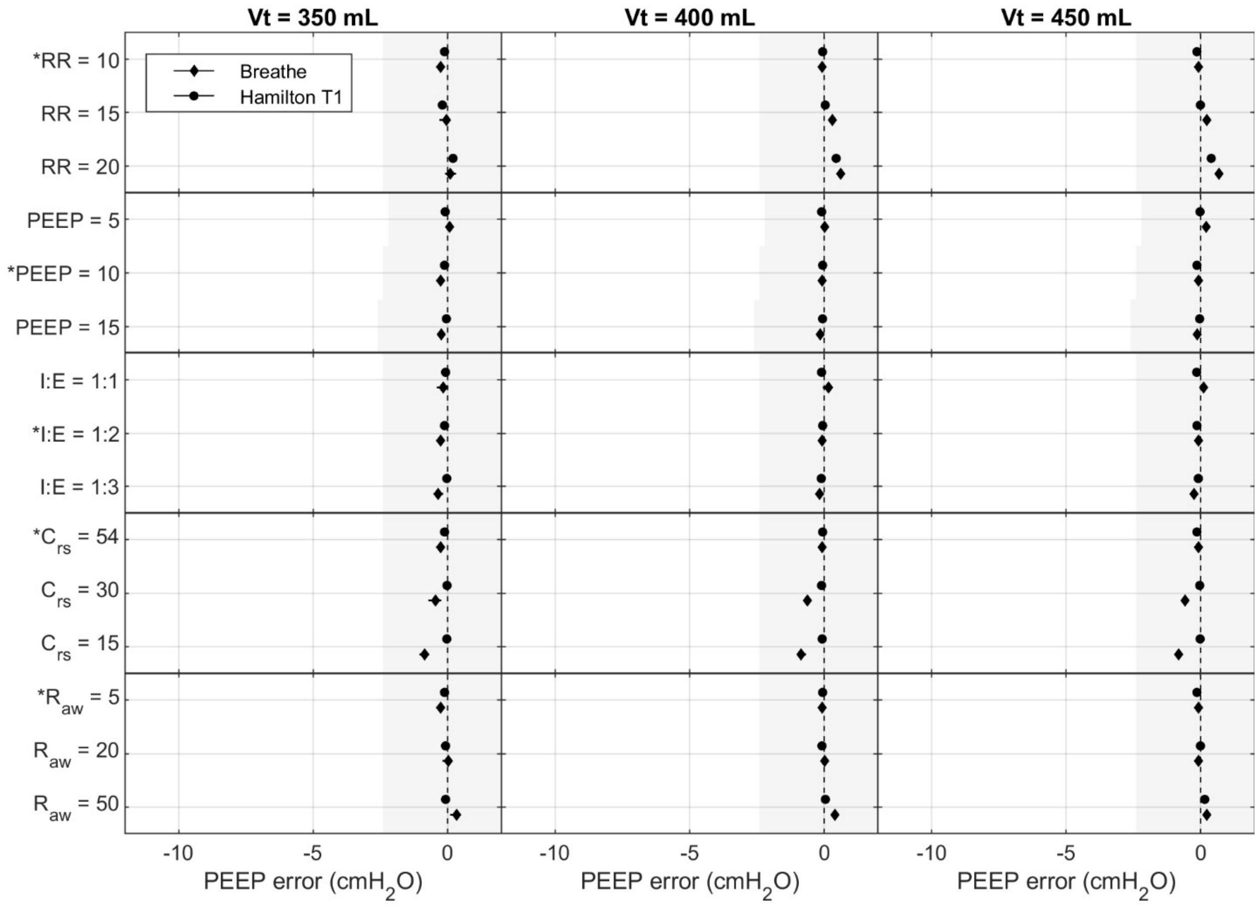

Supplementary Figure 8 Measured PEEP error in the Hamilton T1 ((S)CMV+ mode) and the Breathe: effects of altering V_t (mL), RR (bpm), PEEP (cmH_2O), I:E (ratio), C_{rs} (mL/cmH_2O) and R_{aw} ($cmH_2O/(L/s)$). Base setting indicated by *. The markers and lines denote the mean error and the maximum and minimum errors, respectively. The grayed-out area defines the acceptable performance (ISO norm).

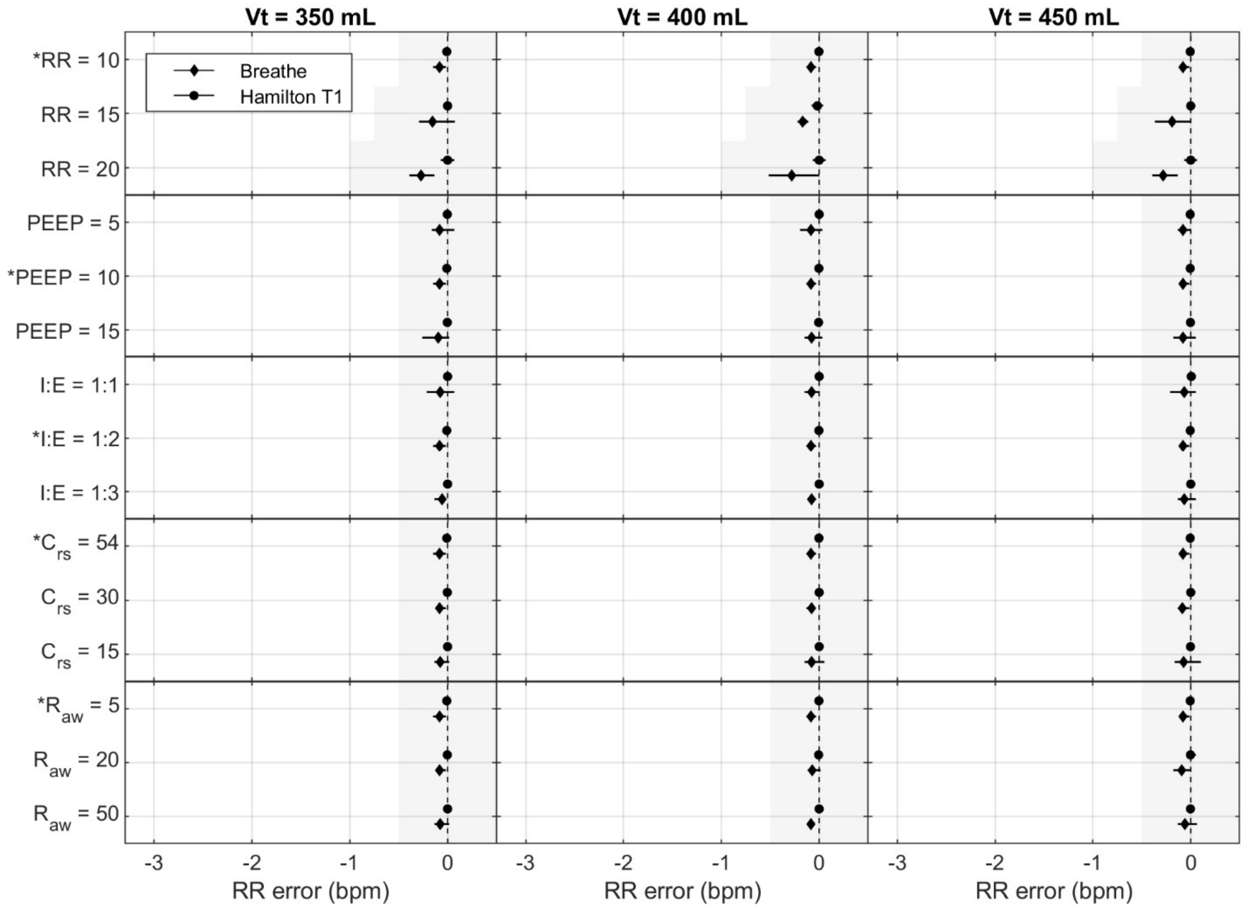

Supplementary Figure 9 Measured RR error in the Hamilton T1 ((S)CMV+ mode) and the Breathe: effects of altering V_t (mL), RR (bpm), PEEP (cmH_2O), I:E (ratio), C_{rs} (mL/cmH_2O) and R_{aw} ($cmH_2O/(L/s)$). Base setting indicated by *. The markers and lines denote the mean error and the maximum and minimum errors, respectively. The grayed-out area defines the acceptable performance (±5%).

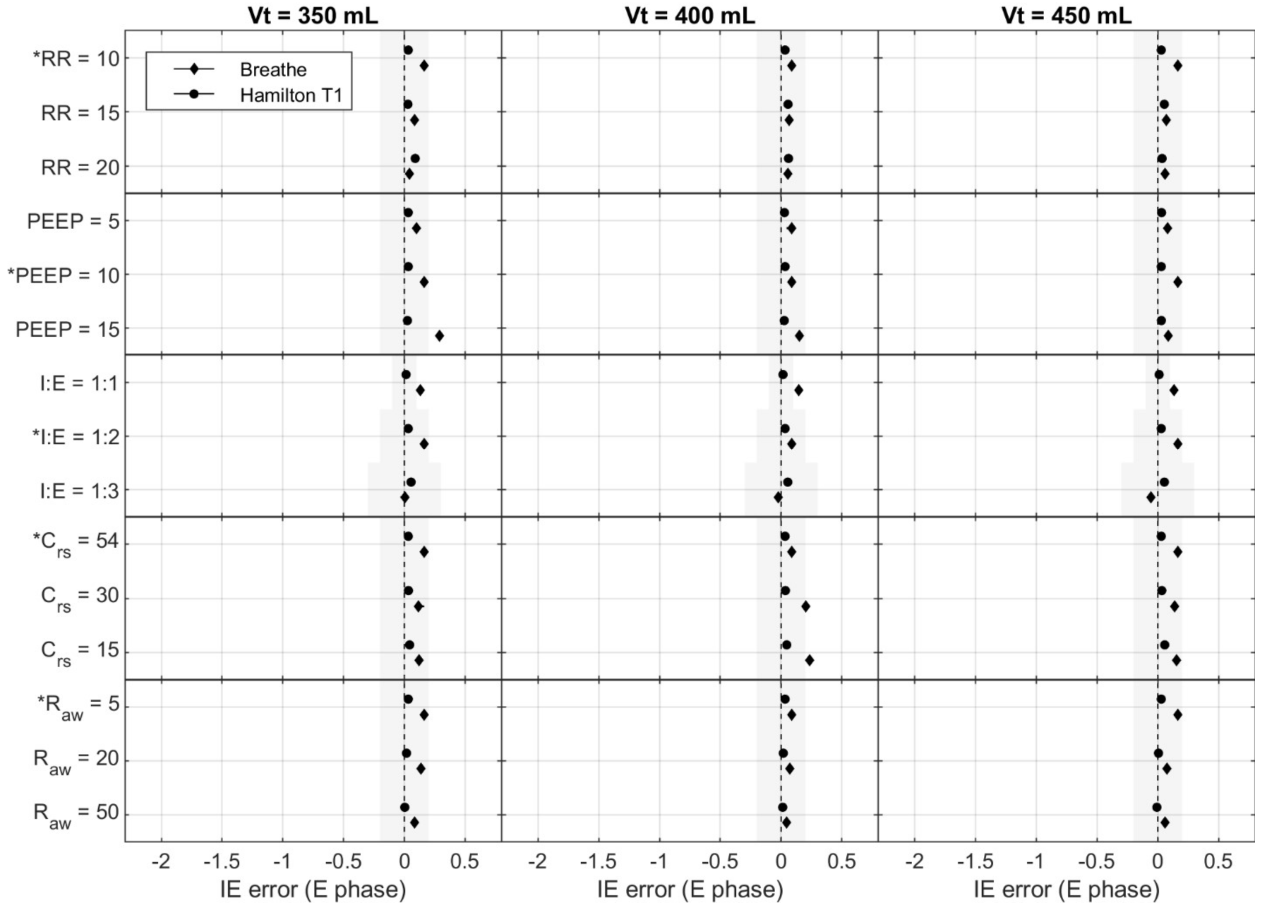

Supplementary Figure 10 Measured I:E error in the Hamilton T1 ((S)CMV+ mode) and the Breathe: effects of altering V_t (mL), RR (bpm), PEEP (cmH_2O), I:E (ratio), C_{rs} (mL/cmH_2O) and R_{aw} ($cmH_2O/(L/s)$). Base setting indicated by *. The markers and lines denote the mean error and the maximum and minimum errors, respectively. The grayed-out area defines the acceptable performance (±10%).

4 Flow profiles

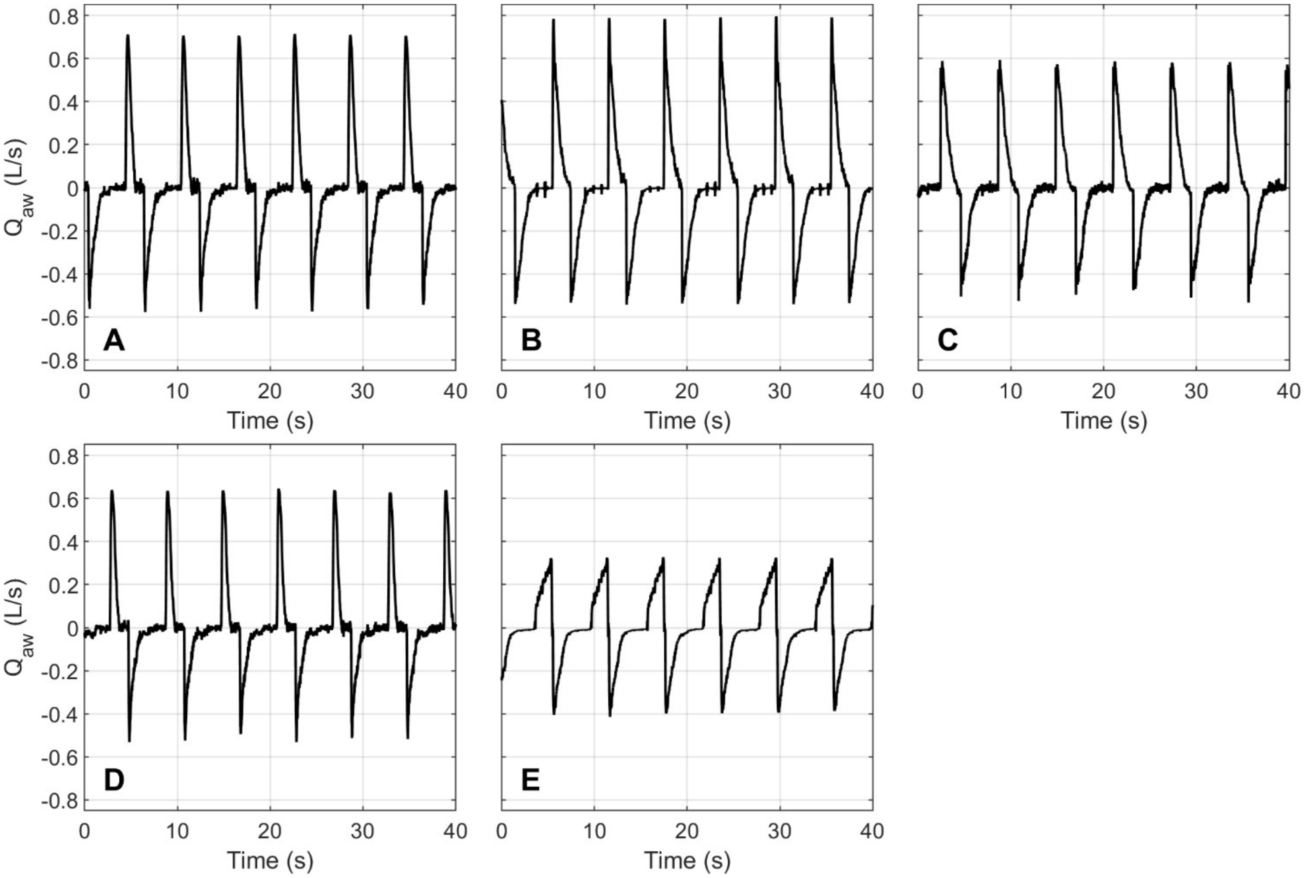

Supplementary Figure 11 Measured flow profiles in the base setting for all ventilators tested (PIP 20cmH_2O or Vt 350mL, RR = 10 bpm, PEEP = 10 cmH_2O, I:E = 1:2). A) Hamilton T1 PCV+ mode, B) HEV Cern, C) GirVent, D) Hamilton T1 (S)CMV mode, E) breathe.

5 Efficiency of oxygen use and response to a change in oxygen supply

5.1 Overview

Supplementary Table 3 Mean, minimum and maximum measured FiO_2 at three O_2 flow rates (2 L/min, 4 L/min and 6 L/min) for the ventilators with low-pressure O_2 supply. Mean, minimum and maximum O_2 concentration are depicted over the last 30s of steady state.

	FiO_2 [%]								
V_{O2} (L/min)		2			4			6	
Ventilator	Min	Mean	Max	Min	Mean	Max	Min	Mean	Max
Hamilton T1	41.5	51.8	57.7	64.0	73.7	80.3	74.8	83.0	88.3
GirVent-LP1	27.0	27.3	28	32.8	33.3	34.7	38.1	38.9	41
GirVent-LP2	24.5	25.5	29.5	28.3	30	35.6	32.1	37.3	53.8
Breathe	48.6	48.8	49.2	78.5	78.8	79.4	100	100	100

Supplementary Table 4 Measured O_2 concentrations of the Hamilton T1 and HEV (high-pressure port) at set concentrations in the UI ((40%, 60%, 80% and 100%).

	FiO_2 [%]											
FiO_2 set (%)		40			60			80			100	
Ventilator	Min	Mean	Max	Min	Mean	Max	Min	Mean	Max	Min	Mean	Max
Hamilton T1	39.1	39.6	40.4	60.1	60.7	61.8	78.6	79.5	81.3	100	100	100
HEV Cern	58	59.6	60.7	75.7	77	78.4	92	94.7	97	100	100	100

Supplementary Table 5 $FiO_{2,t90}$ and t90 at O_2 flow rate of 12 L/min (low-pressure) or a set O_2 concentration of 100% (high-pressure) of each ventilator.

	Low-pressure		High-pressure	
Parameter	$FiO_{2,t90}$ [%]	t_{90} [s]	$FiO_{2,t90}$ [%]	t_{90} [s]
O_2 supply / setting	12 (L/min)	12 (L/min)	100 (%)	100 (%)
	Mean	Mean	Mean	Mean
T1	88.9	135.9	92.1	125.3
GirVent – LP1	50.9	105.9	-	-
GirVent – LP2	42.6	123.4	-	-
Breathe	92.1	260.8	-	-
HEV	-	-	92.1	213.3

5.2 O_2 High-Pressure Port

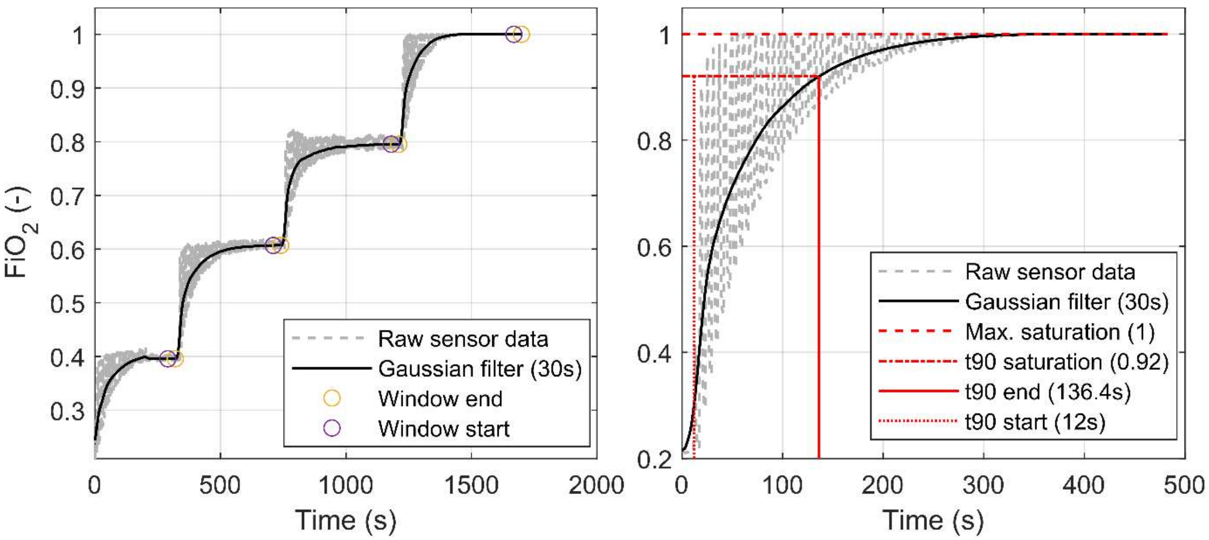

Supplementary Figure 12 Oxygen dynamics tests for the Hamilton T1 (high pressure port). The results are based on the last 30s before changing the O_2 supply or setting (time window indicated). Left: FiO_2 measured at different O_2 concentration set in the ventilator user interface (40%, 60%, 80% and 100%). Right: measurement of the t_{90} time of the high-pressure port with an O_2 setting of 100% in the user interface.

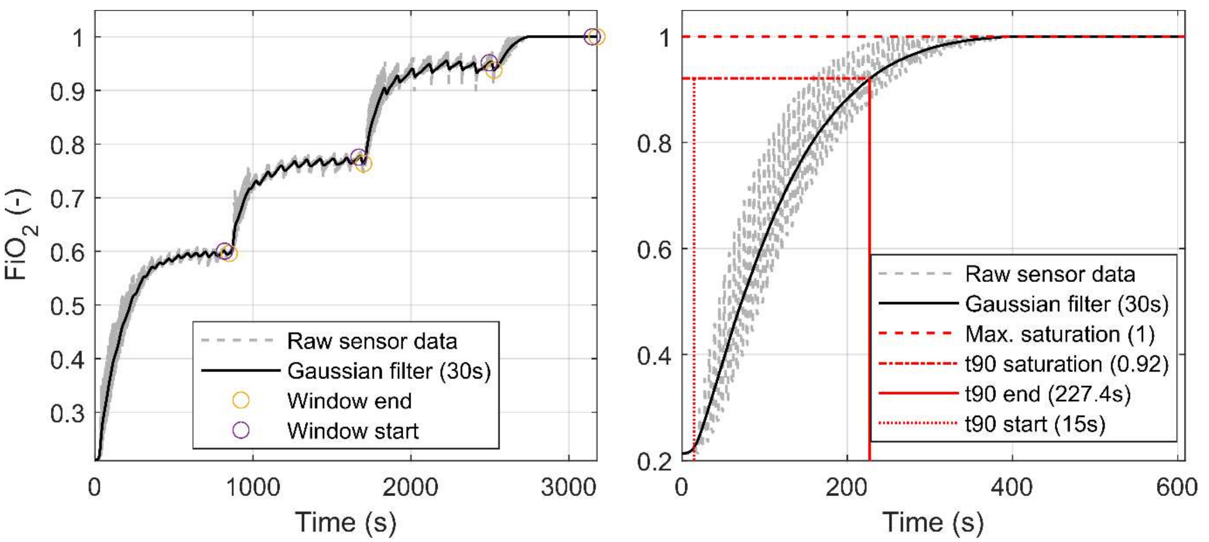

Supplementary Figure 13 Oxygen dynamics tests for the HEV (high pressure port). The results are based on the last 30s before changing the O_2 supply or setting (time window indicated). Left: FiO_2 measured at different O_2 concentration set in the ventilator user interface (40%, 60%, 80% and 100%). Right: measurement of the t_{90} time of the high-pressure port with an O_2 setting of 100% in the user interface.

5.3 O_2 Low-Pressure Port

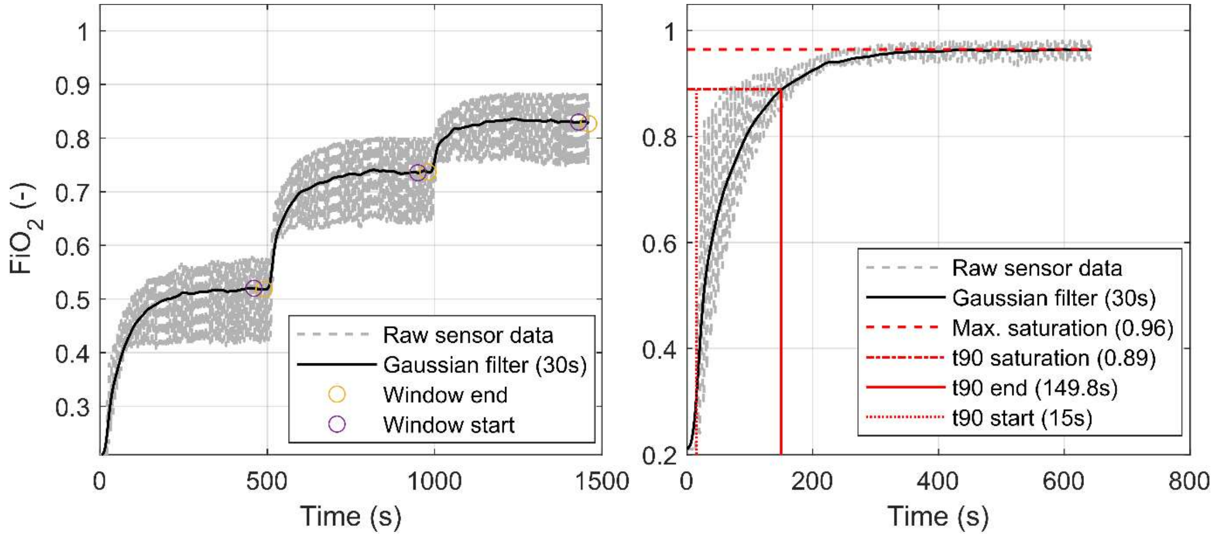

Supplementary Figure 14 Oxygen dynamics tests for the Hamilton T1 (low pressure port). The results are based on the last 30s before changing the O_2 supply or setting (time window indicated). Left: FiO_2 measured of the low-pressure port at an oxygen supply flow rate of 2L/min, 4L/min and 6L/min. Right: measurement of the t_{90} time of the low-pressure port with an O_2 supply of 12L/min.

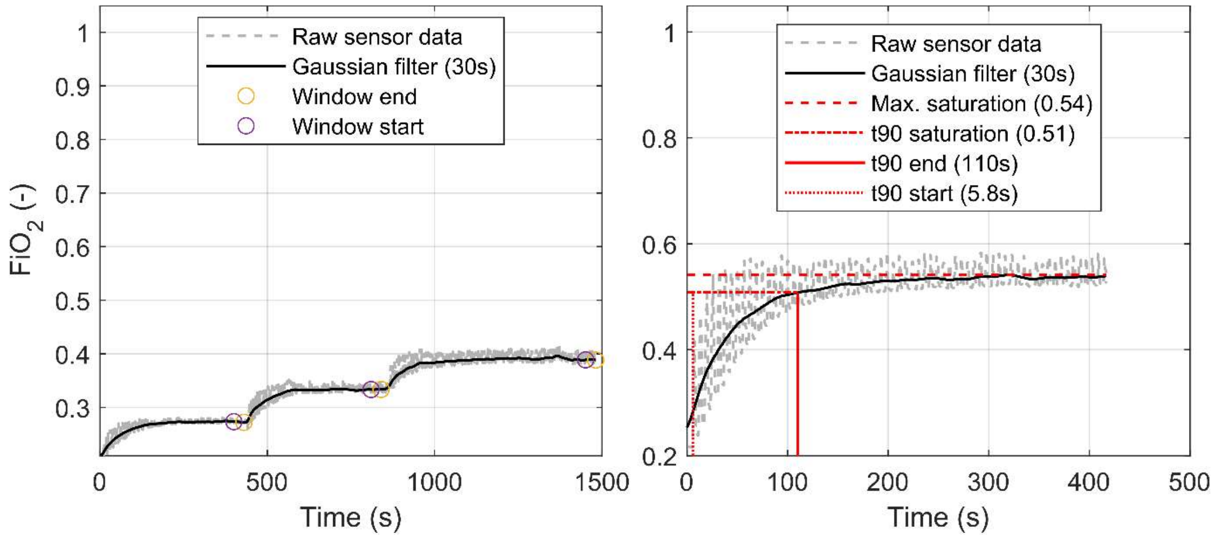

Supplementary Figure 15 Oxygen dynamics tests for the GirVent (low pressure port 1). The results are based on the last 30s before changing the O_2 supply or setting (time window indicated). Left: FiO_2 measured of the low pressure port at an oxygen supply flow rate of 2L/min, 4L/min and 6L/min. Right: measurement of the t_{90} time of the low pressure port with an O_2 supply of 12L/min.

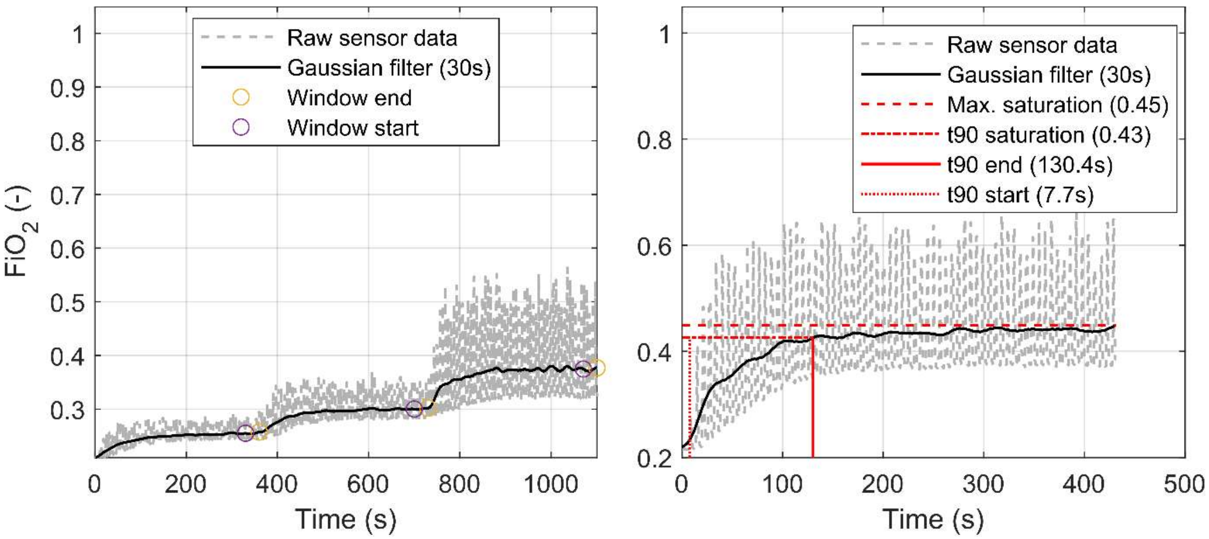

Supplementary Figure 16 Oxygen dynamics tests for the GirVent (low pressure port 2). The results are based on the last 30s before changing the O_2 supply or setting (time window indicated). Left: FiO_2 measured of the low-pressure port at an oxygen supply flow rate of 2L/min, 4L/min and 6L/min. Right: measurement of the t_{90} time of the low-pressure port with an O_2 supply of 12L/min.

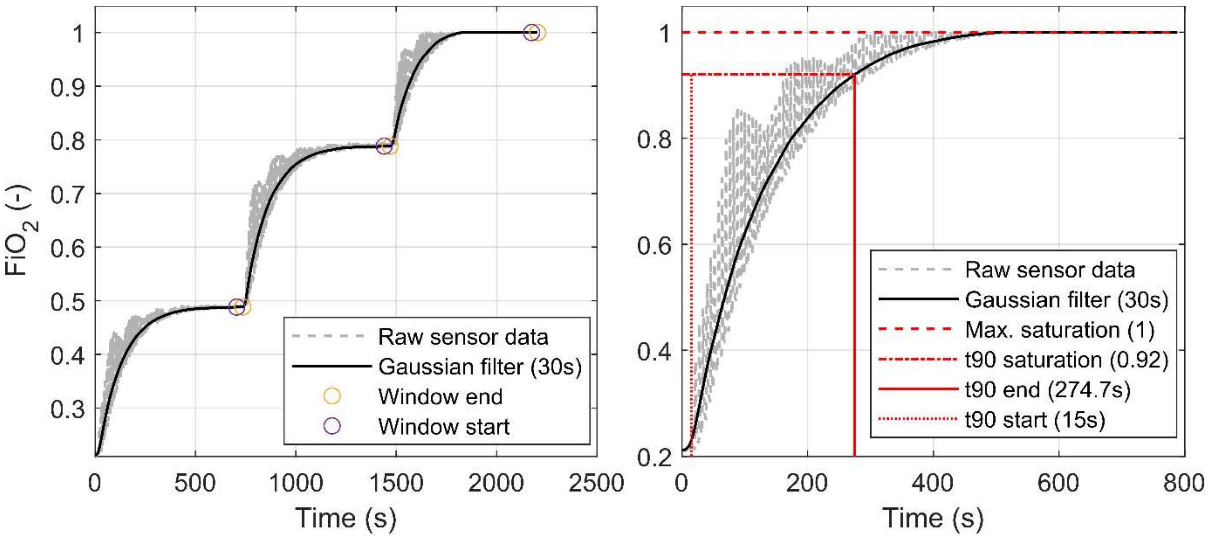

Supplementary Figure 17 Oxygen dynamics tests for the Breathe (low pressure port). The results are based on the last 30s before changing the O_2 supply or setting (time window indicated). Left: FiO_2 measured of the low-pressure port at an oxygen supply flow rate of 2L/min, 4L/min and 6L/min. Right: measurement of the t_{90} time of the low-pressure port with an O_2 supply of 12L/min.

6 Ventilator Trigger Signals

Supplementary Table 6 Mean values and standard deviation (SD) of trigger signals pressure drop (PD), trigger delay time (TDT), time to pressure minimum (TPM) and pressure time product during trigger (PTP) of each ventilator in assisted breathing at a patient breathing gradient of 2 cmH_2O/100 ms.

Trigger signals	PD (cmH_2O)	TPM (s)	TDT (s)	PTP (cmH_2O*s)	PC300 (%)	PC500 (%)
	Mean ± SD	Mean ± SD	Mean ± SD	Mean ± SD	Mean ± SD	Mean ± SD
Hamilton T1	1.20 ± 0.10	0.09 ± 0.01	0.24 ± 0.03	0.17 ± 0.03	51.58 ± 7.04	67.66 ± 4.55
GirVent	3.68 ± 0.47	0.26 ± 0.02	0.33 ± 0.02	0.69 ± 0.09	40.46 ± 3.61	52.84 ± 3.83
Breathe	5.80 ± 0.17	0.28 ± 0.01	0.49 ± 0.02	1.28 ± 0.07	9.64 ± 0.94	15.67 ± 0.92
HEV	1.74 ± 0.08	0.11 ± 0.01	0.17 ± 0.01	0.15 ± 0.01	24.72 ± 1.20	42.00 ± 1.00

Supplementary Table 7 Mean values and standard deviation (SD) of trigger signals pressure drop (PD), trigger delay time (TDT), time to pressure minimum (TPM) and pressure time product during trigger (PTP) of each ventilator in assisted breathing at a patient breathing gradient of 4 cmH_2O/100 ms.

Trigger signals	PD (cmH_2O)	TPM (s)	TDT (s)	PTP (cmH_2O*s)	PC300 (%)	PC500 (%)
	Mean ± SD	Mean ± SD	Mean ± SD	Mean ± SD	Mean ± SD	Mean ± SD
Hamilton T1	4.23 ± 0.19	0.28 ± 0.01	0.32 ± 0.01	0.90 ± 0.03	69.48 ± 0.87	80.17 ± 0.68
GirVent	7.35 ± 0.29	0.28 ± 0.02	0.35 ± 0.01	1.50 ± 0.06	51.17 ± 1.63	64.07 ± 1.40
Breathe	11.56 ± 0.11	0.28 ± 0.00	0.51 ± 0.00	2.69 ± 0.06	5.48 ± 0.32	10.85 ± 0.37
HEV	5.77 ± 0.04	0.28 ± 0.00	0.32 ± 0.00	0.96 ± 0.01	61.72 ± 0.45	72.53 ± 0.40
