# Supplementary material for: Testing of pandemic ventilators under early and agile development
Source: Front Med Technol. 2022 Aug 16;4:899328. doi: 10.3389/fmedt.2022.899328 (PMC9424737; doi:10.3389/fmedt.2022.899328)
Supplement: Supplementary file 2 [file Data_Sheet_1.PDF]

Manual – Testing and evaluation of PDVs in early development

- Why this tool
  - The presented testing protocol and the TestChest in combination with the SFM3019 allow for an unbiased and reproducible testing. In order to evaluate the testing data in a fast and also unbiased manner, which would only be partially given in a manual evaluation of the data, we developed automated evaluation scripts for the data produced by TestChest and the SFM3019.
    The tool generates several outputs:
    - A .mat file, including all the values of the metrics that have been identified
    - An Excel table to make the values from the .mat file more presentable and to evaluate if the values are within the tolerances
    - Figures that present the .mat data as line plots of the mean, maximum and minimum error in each test scenario.

- How to use it
  - The automatic evaluation scripts are specifically designed for the test setup as described in the manuscript. The TestChest as a mechanical lung and a Sensirion flow sensor were used. Another setup might be used if either the import data is structured the same as with the TestChest (.csv columns) or if the used data struct in MATLAB has the same parameter entries (naming).
  - The plotting of the data for the accuracy of the controls and systems is optimized for the 33 measurements described in the manuscript. If more measurements will be performed, the script needs to be adjusted accordingly.
  - Three different main scripts are available for evaluating and plotting the accuracy of the controls and systems, the oxygen dynamics and the trigger signals

Manual – Folder structure

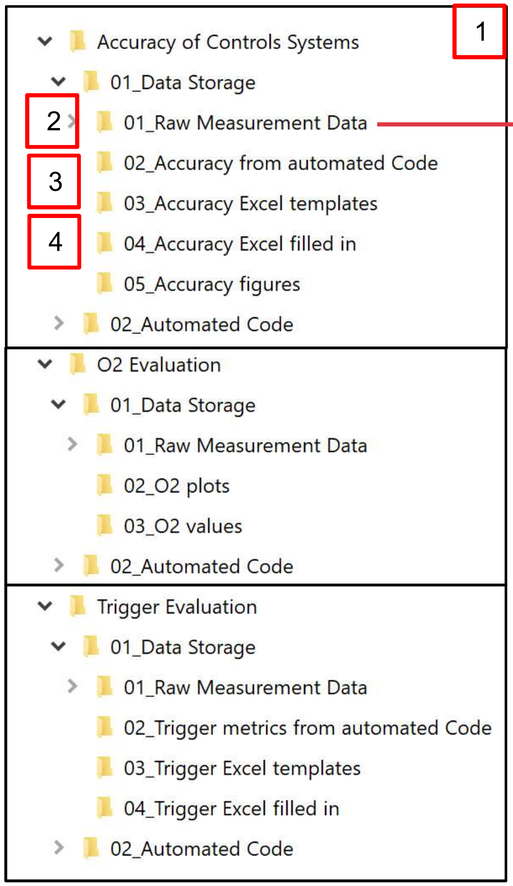

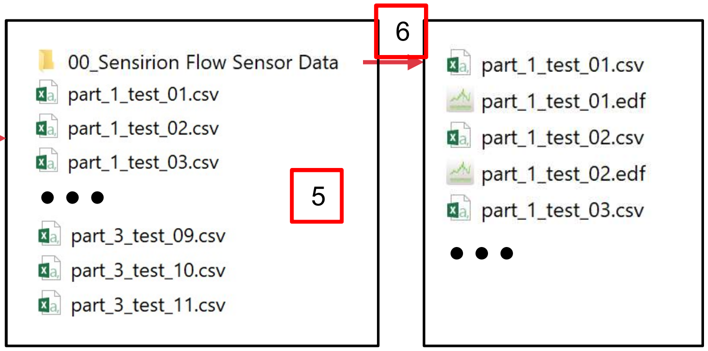

1. Make sure to have the folder structure on the left.
2. Go to *01_Raw Measurement Data.*
3. Create a new folder with the name of the ventilator.
4. Create a subfolder in the ventilator folder, consisting of the ventilator name and the versioning.
   → *ventilatorname*_vX, where X is an increasing number starting at 1.
5. Inside *ventilatorname*_vX folder, place the TestChest .csv data for the 33 experiments
6. If there is also Sensirion SFM3019 log data, create a folder *00_Sensirion Flow Sensor Logging*. Copy the .edf files recorded with Sensirion Control Center inside this folder.
7. An example of the folder structure for the three evaluations is given in the provided example

Manual – Data structure TestChest output

part_1_test_01.csv
part_1_test_02.csv
part_1_test_03.csv

The TestChest will generate the following column structure in the csv output files (delimiter semicolon). It is crucial to maintain this structure for the presented MATLAB scripts to run properly.

Time | Flow | Lung tidal volume | p alveole | Airway pressure | Bellows position | Intrapleural pressure | Cardial pressure | Fraction of O2 inspir | Ambient pressure | Temperature Celsius | FRC actual | Shunt

Time | Flow | Lung tidal volume | ~~p alveole~~ | ~~Airway pressure~~ | ~~Bellows position~~ | Intrapleural pressure | ~~Cardial pressure~~ | Fraction of O2 inspir | ~~Ambient pressure~~ | ~~Temperature Celsius~~ | ~~FRC actual~~ | Shunt

For the evaluation of the accuracy of the controls and systems, the oxygen dynamics and the trigger signals only the above columns in the exported data is needed. In case you use another mechanical lung or recording system, keep the column structure as follows or change the import function in the MATLAB file *readventilator.m*

```
function data_struct = readVentilatorCSV(csvfolder,Fs)
%% Go to csv location
cd(csvfolder)

%% Make csv file list
ms = dir('*.csv');

%% Loop over file list and import data
for i = 1:length(ms)
    disp(strcat("Load csv: ",num2str(i),'/',num2str(length(ms))))
    filename = ms(i).name;
    data_read = importfileTC(filename,Fs);
    data_struct_temp = table2struct(data_read,'ToScalar',true);
    data_struct_temp.csvname = filename;
    data_struct(i) = data_struct_temp;
    clear data_struct_temp
    clear data_read
end

end %function
```

Manual – Accuracy of controls and systems

```matlab
%% Run the Accuracy Evaluation, write results in Excel and plot Figures
% Nicola Steffen, pdz 2020
clear
clc
close all
cd(fileparts(which(mfilename)));   % cd to this script's location
%% User input
% Write the session name, which must correspond to the folder name in
% 01_Data Storage holding the raw measurement data from TestChest and
% optionally the SFM3019 flow sensor. If there are several test sessions
% for a specific ventilator, specify which version you want to evaluate
sessionname     = ["Hamilton T1"];
version         = ["v2"];
Fs              = 100; % Specify sampling frequency in Hz, 50Hz for TestChest V2, 100Hz for V3
xlsxtemplate    = "Accuracy_template_VolumeControl_minmax_v1.xlsx"; % Specify which .xlsx template to use for the Excel file
% xlsxtemplate    = "Accuracy_template_PressureControl_minmax_v1.xlsx"; % Specify which .xlsx template to use for the Excel file
% typeofcontrol   = ["pressure"]; % either "volume" or "pressure"
typeofcontrol   = ["volume"]; % either "volume" or "pressure"

plotting        = "off";        % "continous" plots each experiment as fast as possible
                                % "single" stops at every experiment and waits for the user to press a key
                                % "off" suppresses plotting (faster execution);
plotunfiltered  = "off";     % "on" or "off", plots unfiltered data under the filtered data if plotting is active
%% Automated evaluation (no change necessary)
addpath('00_Functions')
AccuracyEvaluation(sessionname,Fs,version,plotting,plotunfiltered)
Accuracy2Excel(sessionname,version,xlsxtemplate,typeofcontrol)
for i = 1:length(sessionname)
    Accuracy2ErrorbarsMultiBar(sessionname(i),version(i),typeofcontrol(i),i)
end
%% Wrap up
% close all;
disp(strcat("Automated Accuracy evaluation for ",sessionname,"_",version," was successful"))
disp("Find the filled in Excel sheet in ../01_Data Storage/04_Accuracy Excel filled in")
disp("Find the Boxplots in ../01_Data Storage/05_Accuracy figures")
```

1. Go to *Standalone Accuracy Eval → 02_Automated Code*
2. Open *run_main.m* (MATLAB version R2020a)
3. Adapt *sessionname* such that it has the same name as the ventilator folder
4. Adapt *version* to have the same ending as the versioning
5. Adapt *Fs* to the recording frequency in Hz of the TestChest (*100*Hz). If you choose the wrong frequency, the script will show an error.
6. Adapt *xlsxtemplate* to the name of the template in *Standalone Accuray Eval → 01_Data Storage → 03_Accuracy Excel templates* you want to use. Make sure to chose a template corresponding to the type of control the ventilator uses (pressure or volume)
7. Adapt *typeofcontrol* to either *pressure* or *volume*, depending on the control type of the ventilator
8. Adapt *plotting* to *single, continuous* or *off*
9. Run the Code

Manual – Accuracy of controls and systems

1. Check the error message, if one occurs, and locate in which function the error occurs

   The most common reason is a wrong naming of the files, folders or a wrong folder structure for the data.

2. For error analysis, open the corresponding function and set a breakpoint right before the error occurs. Check the variables causing the error and the inputs to the function

```
%% Run the Accuracy Evaluation, write results in Excel and plot Figures
% Nicola Steffen, pdz 2020
clear
clc
close all
cd(fileparts(which(mfilename)));   % cd to this script's location
%% User input
% Write the session name, which must correspond to the folder name in
% 01_Data Storage holding the raw measurement data from TestChest and
% optionally the SFM3019 flow sensor. If there are several test sessions
% for a specific ventilator, specify which version you want to evaluate
sessionname     = ["Hamilton T1"];
version         = ["v2"];
Fs              = 100; % Specify sampling frequency in Hz, 50Hz for TestChest V2, 100Hz for V3
xlsxtemplate    = "Accuracy_template_VolumeControl_minmax_v1.xlsx"; % Specify which .xlsx template to use for the Excel file
% xlsxtemplate    = "Accuracy_template_PressureControl_minmax_v1.xlsx"; % Specify which .xlsx template to use for the Excel file
% typeofcontrol   = ["pressure"]; % either "volume" or "pressure"
typeofcontrol   = ["volume"]; % either "volume" or "pressure"

plotting        = "off";        % "continous" plots each experiment as fast as possible
                                % "single" stops at every experiment and waits for the user to press a key
                                % "off" suppresses plotting (faster execution);
plotunfiltered  = "off";     % "on" or "off", plots unfiltered data under the filtered data if plotting is active
%% Automated evaluation (no change necessary)
addpath('00_Functions')
AccuracyEvaluation(sessionname,Fs,version,plotting,plotunfiltered)
Accuracy2Excel(sessionname,version,xlsxtemplate,typeofcontrol)
for i = 1:length(sessionname)
    Accuracy2ErrorbarsMultiBar(sessionname(i),version(i),typeofcontrol(i),i)
end
%% Wrap up
% close all;
disp(strcat("Automated Accuracy evaluation for ",sessionname,"_",version," was successful"))
disp("Find the filled in Excel sheet in ../01_Data Storage/04_Accuracy Excel filled in")
disp("Find the Boxplots in ../01_Data Storage/05_Accuracy figures")
```

Manual – Accuracy of controls and systems

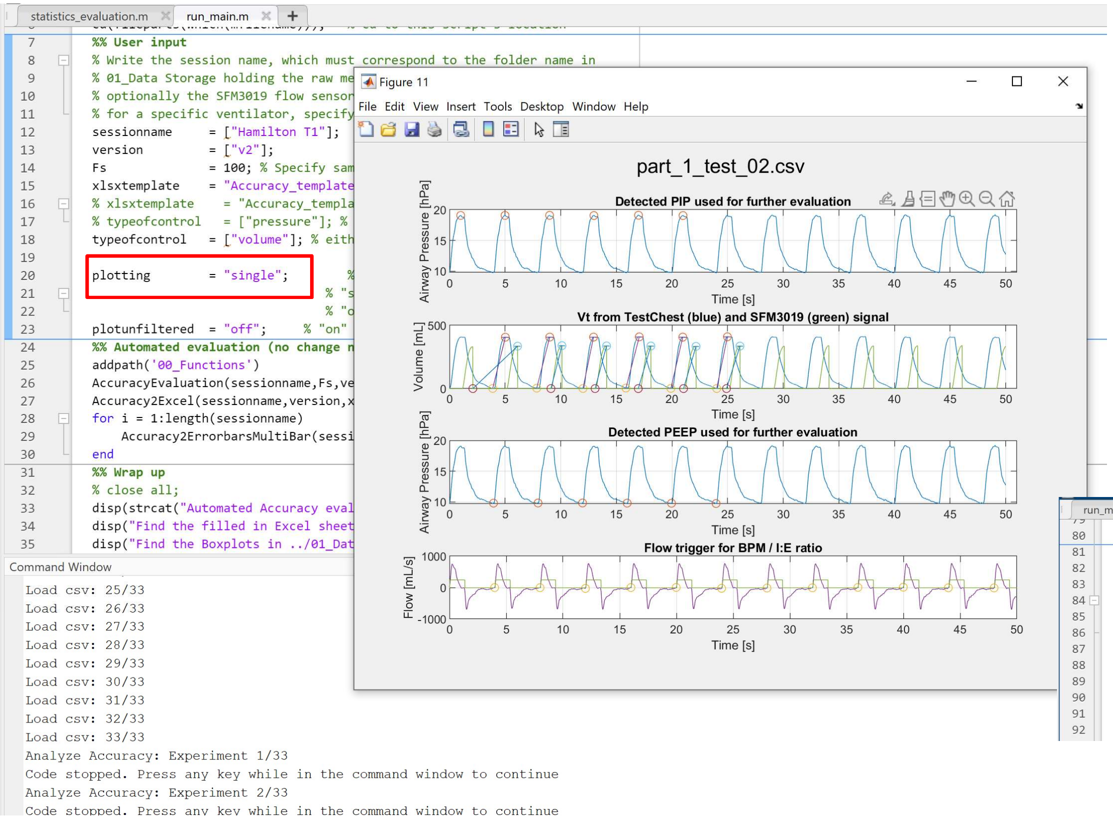

1. In case you select *"single"* as the plotting parameter, all plots of each individual test scenario for the accuracy of controls and systems will be shown in sequence.
2. You can proceed by pressing any key to the next plot of the next experiment.
3. This allow to investigate the detected points for the readout of the values (PIP, PEEP, flow for BPM and I:E ratio).
4. The respective flow threshold for the detection of a flow onset can be adjusted in the function *AccuracyEvaluation.m*

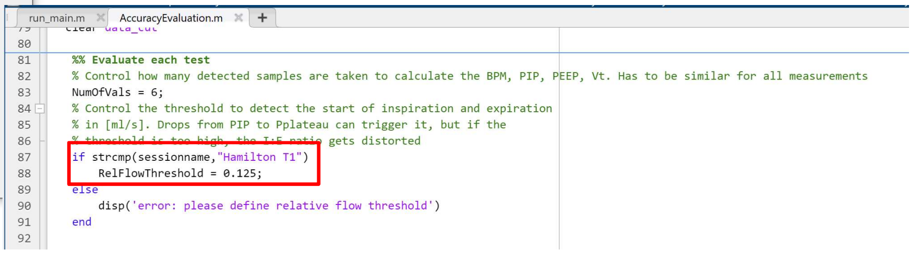

Manual – Accuracy of controls and systems

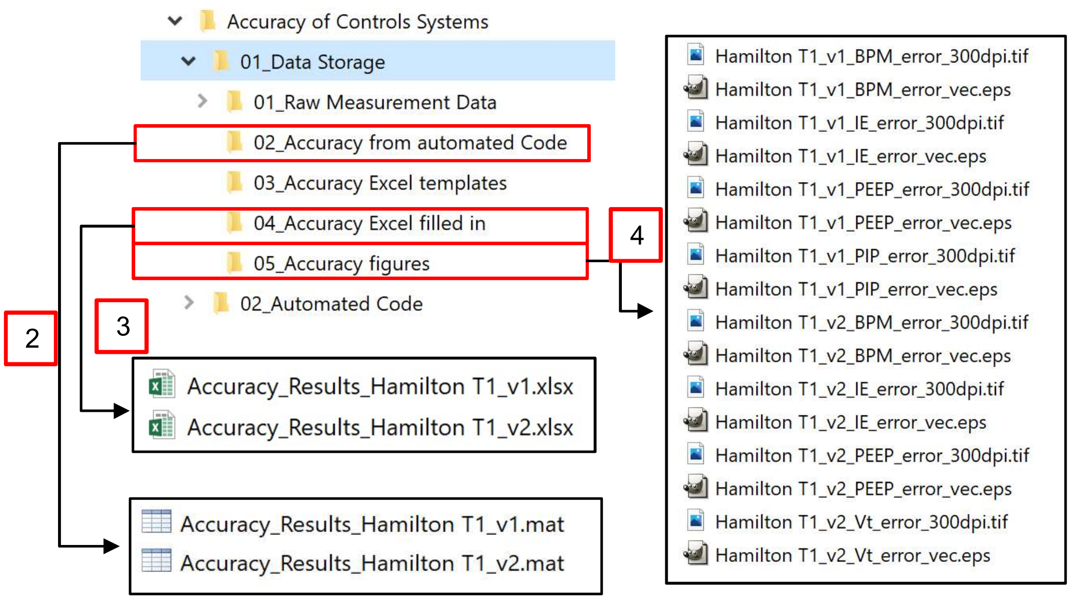

1. After running the script, the following files will be written in the different folders
2. A *.mat* file with the Accuracy results will be written in "02_Accuracy from automated code"
3. An Excel file with the mean, maximum and minimum error of the accuracy tests will be written in "04_Accuracy Excel filled in"
4. All plots of the errors of the different parameters will be created in "05_Accuracy figures"

Manual – O2 efficiency and accuracy

```
1  %% Run the O2 Evaluation, plot Figures
2  % Nicola Steffen, pdz 2020
3  clear
4  clc
5  close all
6  cd(fileparts(which(mfilename)));   % cd to this script's location
7  %% User input
8  % Write the session name, which must correspond to the folder name in
9  % 01_Data Storage holding the raw measurement data from TestChest and
10 % optionally the SFM3019 flow sensor. If there are several test sessions
11 % for a specific ventilator, specify which version you want to evaluate
12 % sessionname     = "GirVent";
13 sessionname     = "Hamilton T1";
14 version         = "v1-HP";
15 Fs              = 100; % Specify sampling frequency in Hz, 50Hz for TestChest V2, 100Hz for V3
16 
17 %% Manual point detection of increased O2 flow
18 % Insert the time in [s] at which the O2 supply was changed (2,4,6L) or % (40%,60%,80%)
19 if strcmp(sessionname,"Hamilton T1") && strcmp(version,"v1-LP")
20     tO2inc = [490,980];
21 end
22 if strcmp(sessionname,"Hamilton T1") && strcmp(version,"v1-HP")
23     tO2inc = [320,740,1210];
24 end
25 
26 %% Automated evaluation (no change necessary)
27 addpath('00_Functions')
28 SteadyStateO2Evaluation(sessionname,Fs,version,tO2inc)
29 t90O2Evaluation(sessionname,Fs,version)
30 %% Wrap up
31 % close all;
32 disp(strcat("Automated O2 evaluation for ",sessionname,"_",version," finished"))
33 disp("Automatic figure saving not implemented ... ")
34 
```

1. Go to *O2 Evaluation* → *02_Automated Code*
2. Open *run_main.m* (Matlab version R2020a)
3. Adapt *sessionname* such that it has the same name as the ventilator folder
4. Adapt *version* to have the same ending as the versioning
5. Adapt *Fs* to the recording frequency in Hz of the TestChest (*100*Hz). If you choose the wrong frequency, the script will show an error.
6. Adapt the time at which the manual O2 change was performed in the high and low pressure tests (line 20 and line 23
7. Run the Code

Manual – O2 efficiency and accuracy plots and data

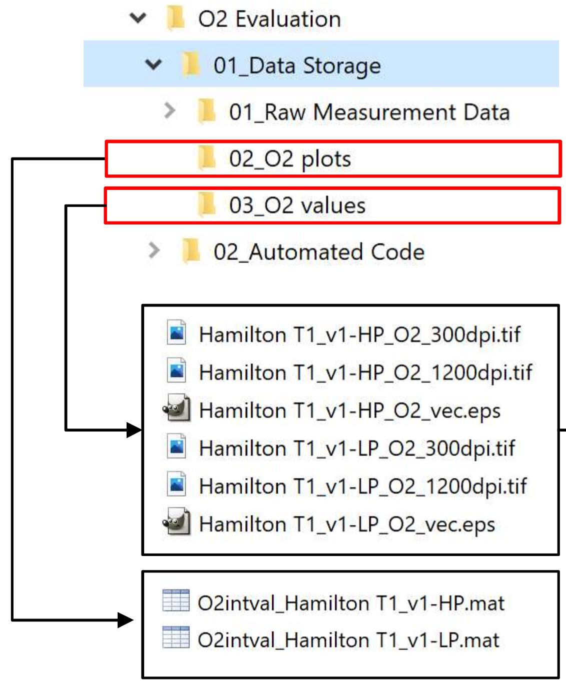

1. The script outputs the following files in the different folders
2. All plots of the oxygen dynamics will be created in “02_O2 plots”
3. A *.mat* file with the oxygen dynamics results will be written in “03_O2 values”

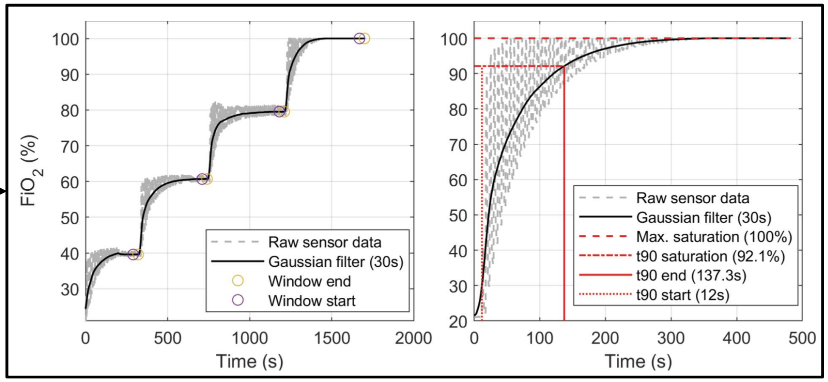

Manual – Trigger signals

```
%% Run the Trigger Evaluation, plot Figures
% Nicola Steffen, pdz 2020
clear
clc
close all
cd(fileparts(which(mfilename)));   % cd to this script's location
%% User input
% Write the session name, which must correspond to the folder name in
% 01_Data Storage holding the raw measurement data from TestChest and
% optionally the SFM3019 flow sensor. If there are several test sessions
% for a specific ventilator, specify which version you want to evaluate
% sessionname     = "Novator";
sessionname     = "Hamilton T1";
version         = "v1_pcv";
xlsxtemplate    = 'Trigger_template_v2.xlsx';
Fs              = 100; % Specify sampling frequency in Hz, 50Hz for TestChest V2, 100Hz for V3
plotting        = "single";  % "continous" plots each experiment as fast as possible
                             % "single" stops at every experiment and waits for the user to press a key
                             % "off" suppresses plotting (faster execution);

%% Automated evaluation (no change necessary)
addpath('00_Functions')
PressureDropEvaluation(sessionname,Fs,version,plotting)
PressureMetricsEvaluation(sessionname,Fs,version,plotting)
Trigger2Excel(sessionname,version,xlsxtemplate)
%% Wrap up
% close all;
disp(strcat("Automated Trigger evaluation for ",sessionname,"_",version," finished"))
```

1. Go to *Trigger Evaluation* → *02_Automated Code*
2. Open *run_main.m* (written in R2020a)
3. Adapt *sessionname* such that it has the same name as the ventilator folder
4. Adapt *version* to have the same ending as the versioning
5. Adapt *Fs* to the recording frequency in Hz of the TestChest (*100*Hz). If you choose the wrong frequency, the script will show an error.
6. Check the *xlsxtemplate* name with the template in *Standalone Trigger Evaluation* → *01_Data Storage* → *03_Trigger Excel templates*
7. Adapt *plotting* to *single* or *off*
8. Run the Code

Manual – Trigger signals

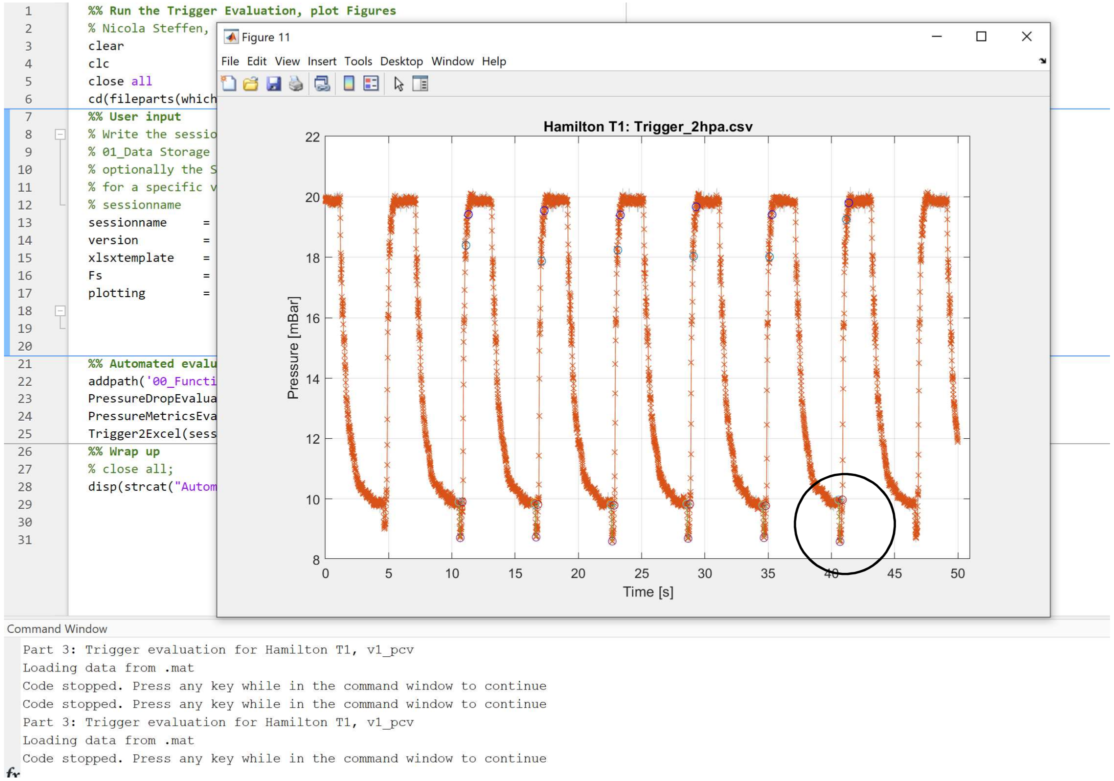

1. After running the code, a figure will show the detected points of the trigger parameters (PD, TPM, TDT, PTP)
2. The first figure will show the plot for P0.1 = 2hPa/100ms. Press any key to continue to the next figure
3. Continue through the individual figures. After that, the data will be stored in *Trigger Evaluation → 01_Data Storage → Trigger Excel filled in*
4. For a more detailed view of the detected points, zoom [illegible]

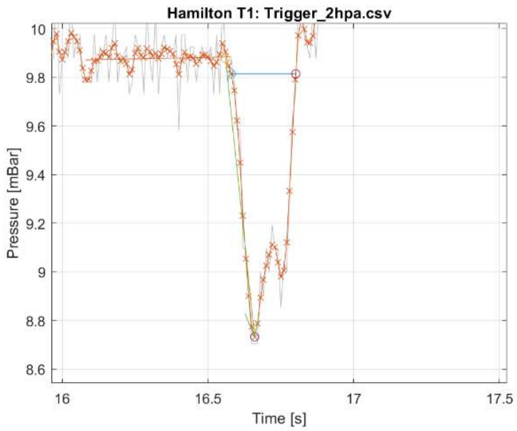

Support – Contact

Marianne Schmid-Daners
Tannenstrasse 3
8092 Zurich
marischm@ethz.ch

Niko Tachatos
Tannenstrasse 3
8092 Zurich
nikolaos.tachatos@alumni.ethz.ch

Mark Zander
Tannenstrasse 3
8092 Zurich
mzander@ethz.ch
