# Supplementary figures and images for: Testing of pandemic ventilators under early and agile development
Source: Front Med Technol. 2022 Aug 16;4:899328. doi: 10.3389/fmedt.2022.899328 (PMC9424737; doi:10.3389/fmedt.2022.899328)

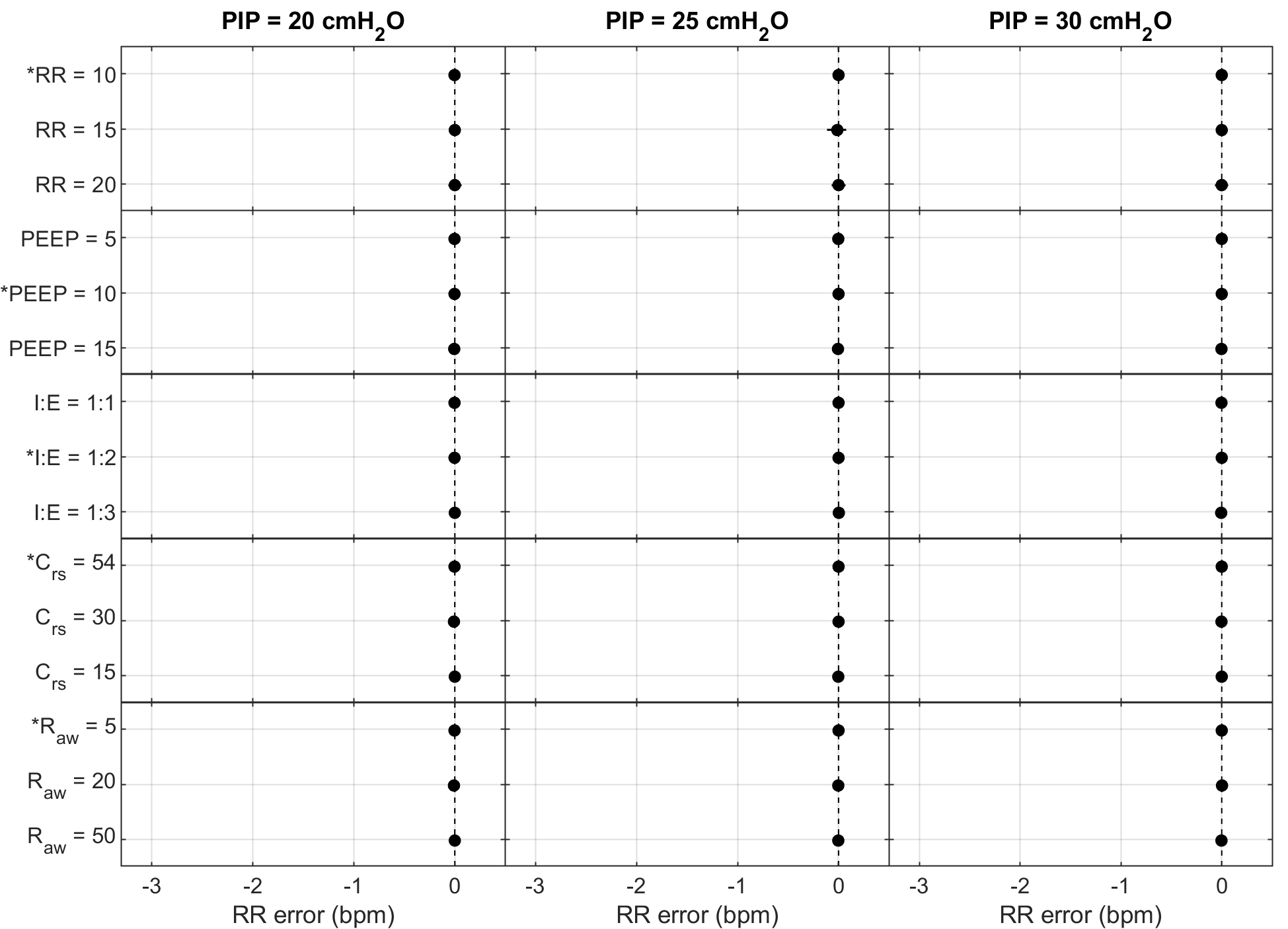

Supplement: Supplementary file 3 [file Data_Sheet_2.ZIP › Accuracy of Controls Systems/01_Data Storage/05_Accuracy figures/Hamilton T1_v1_BPM_error_300dpi.tif]

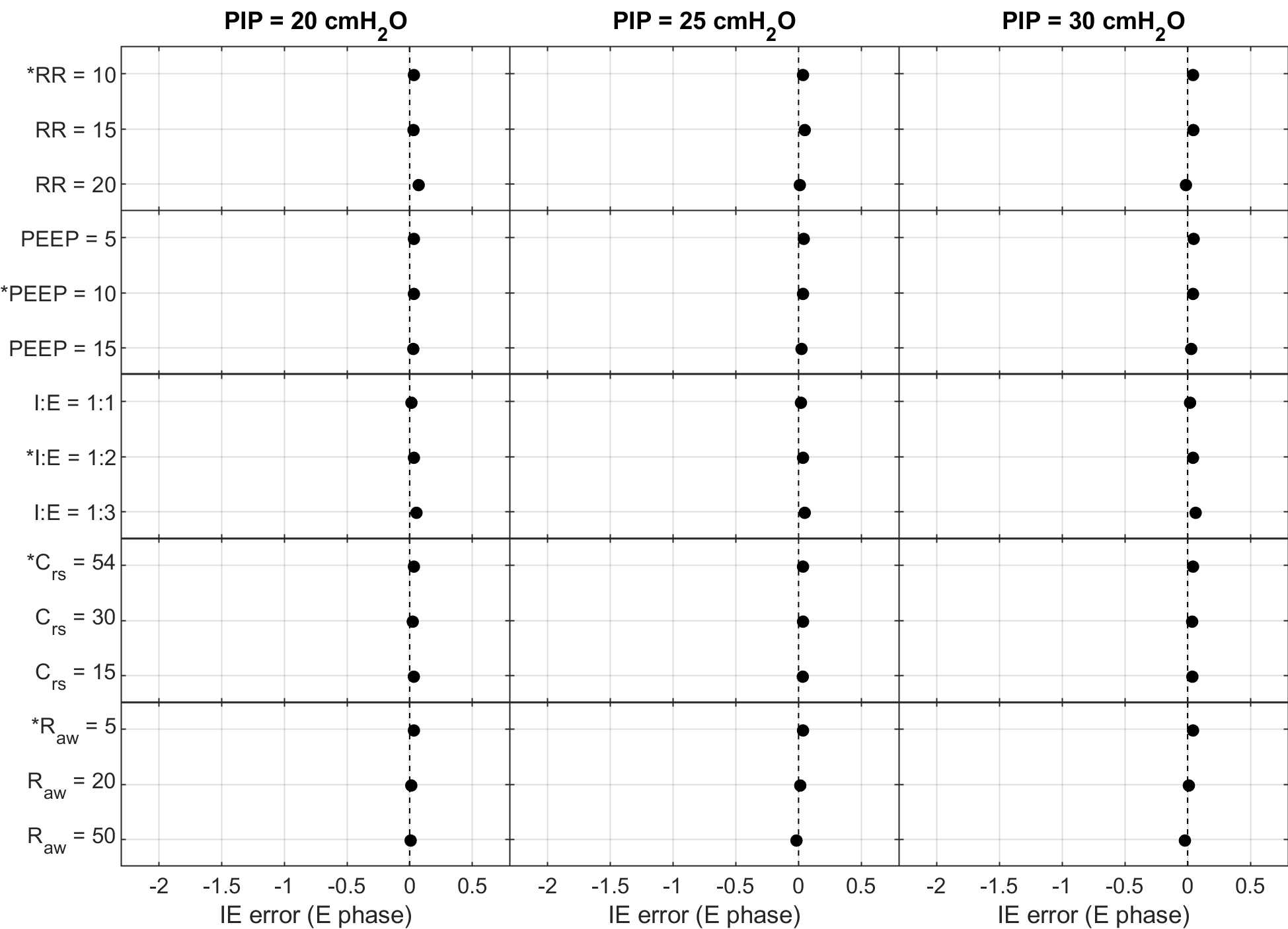

Supplement: Supplementary file 3 [file Data_Sheet_2.ZIP › Accuracy of Controls Systems/01_Data Storage/05_Accuracy figures/Hamilton T1_v1_IE_error_300dpi.tif]

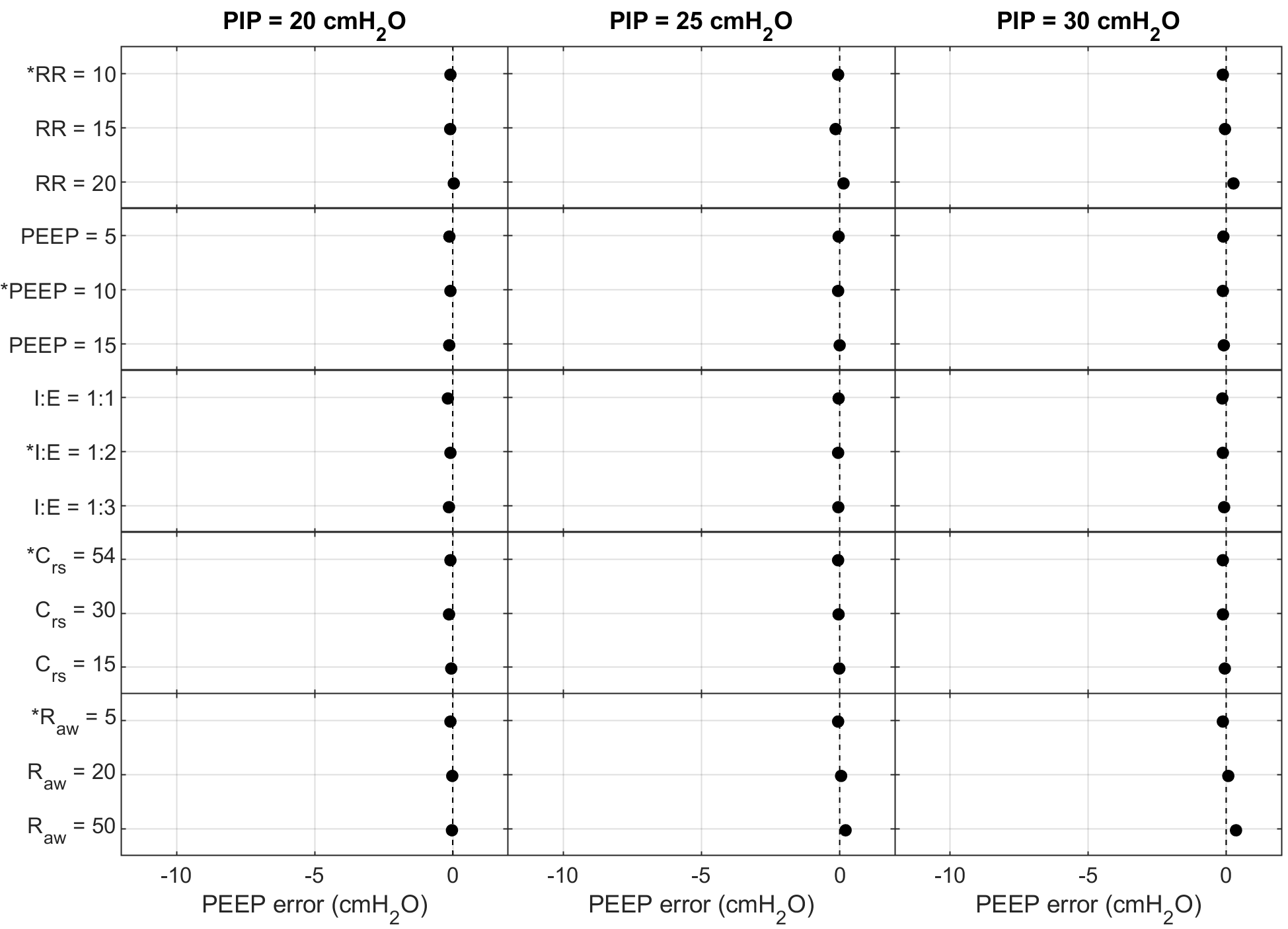

Supplement: Supplementary file 3 [file Data_Sheet_2.ZIP › Accuracy of Controls Systems/01_Data Storage/05_Accuracy figures/Hamilton T1_v1_PEEP_error_300dpi.tif]

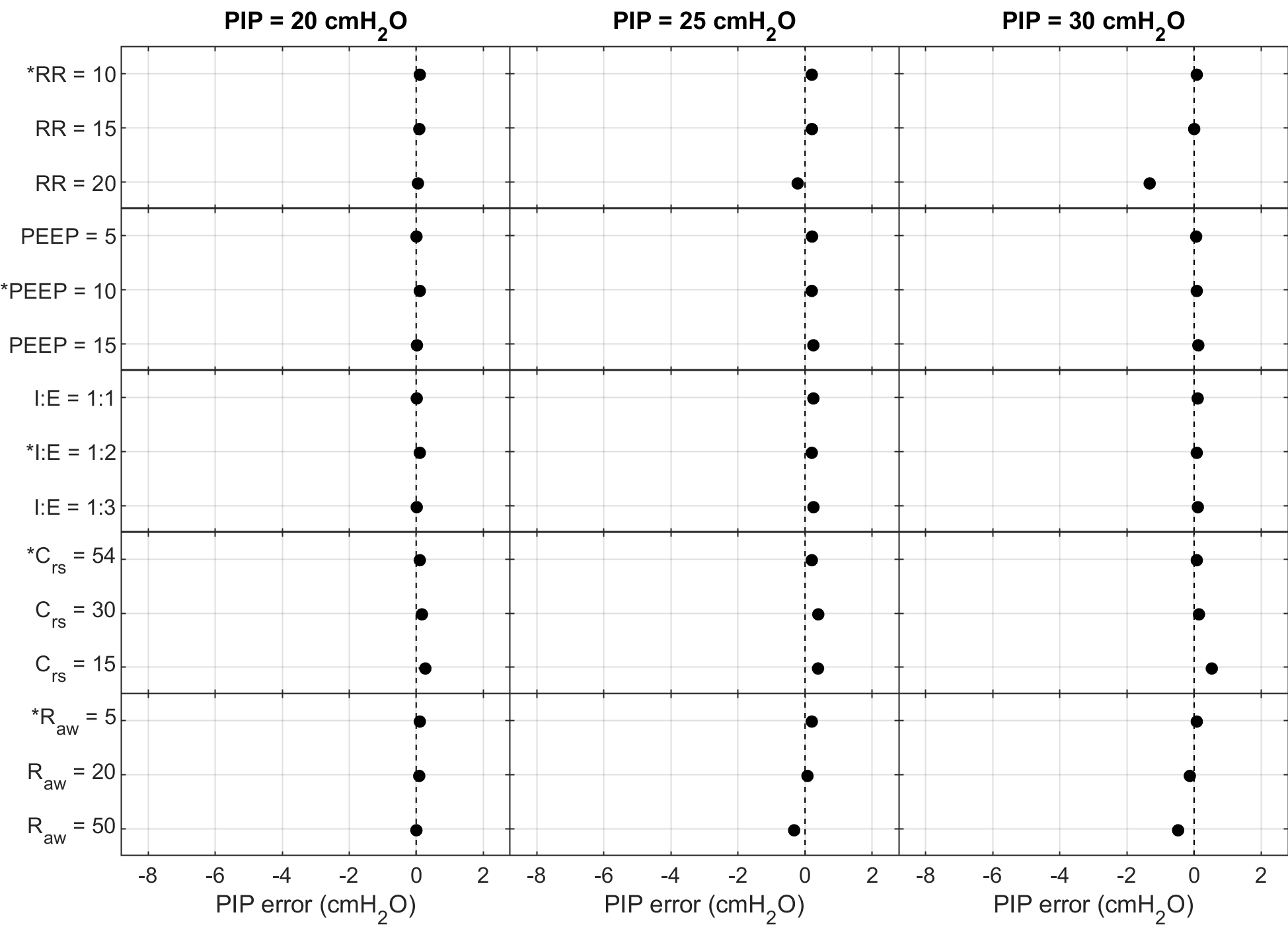

Supplement: Supplementary file 3 [file Data_Sheet_2.ZIP › Accuracy of Controls Systems/01_Data Storage/05_Accuracy figures/Hamilton T1_v1_PIP_error_300dpi.tif]

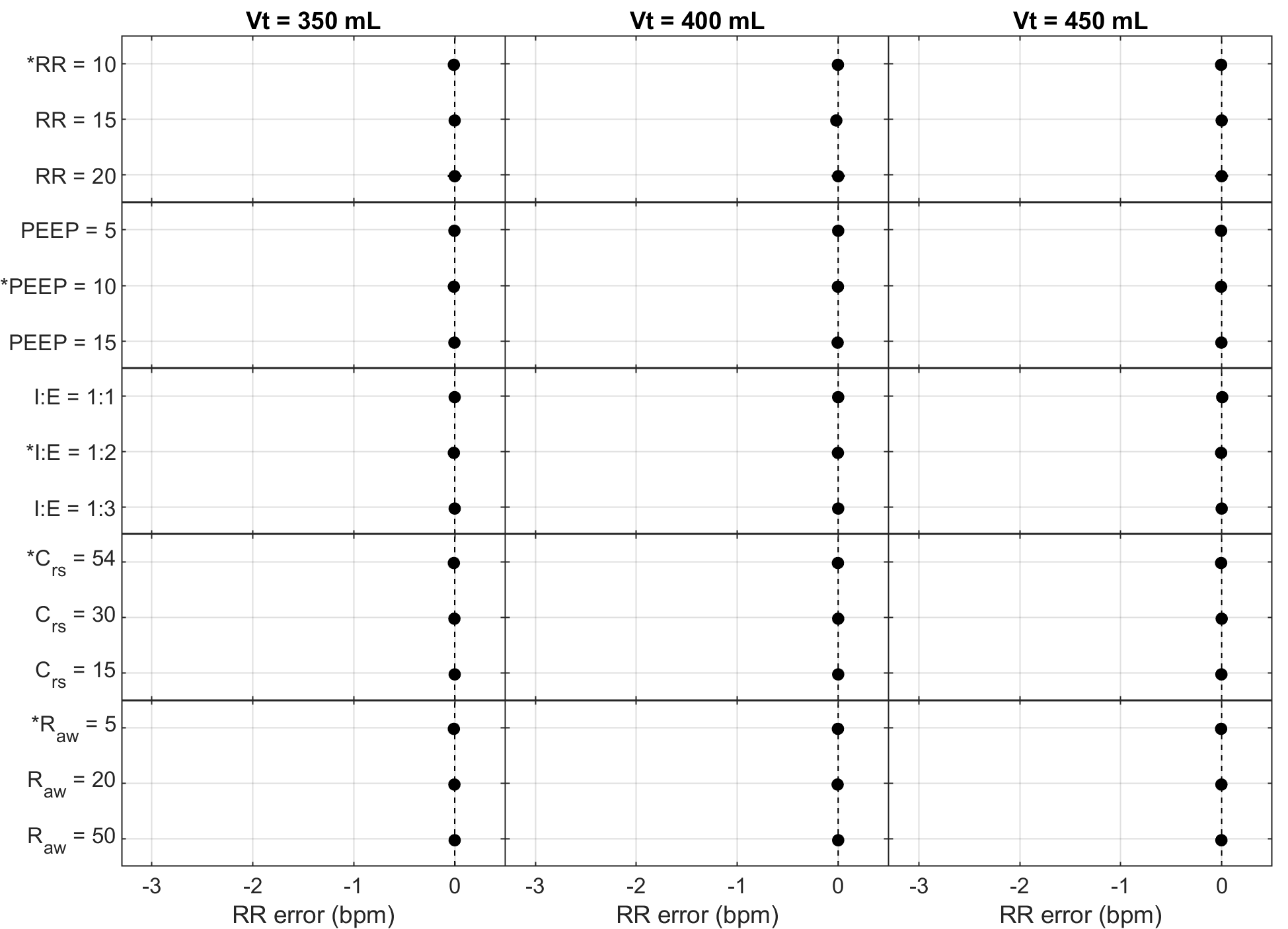

Supplement: Supplementary file 3 [file Data_Sheet_2.ZIP › Accuracy of Controls Systems/01_Data Storage/05_Accuracy figures/Hamilton T1_v2_BPM_error_300dpi.tif]

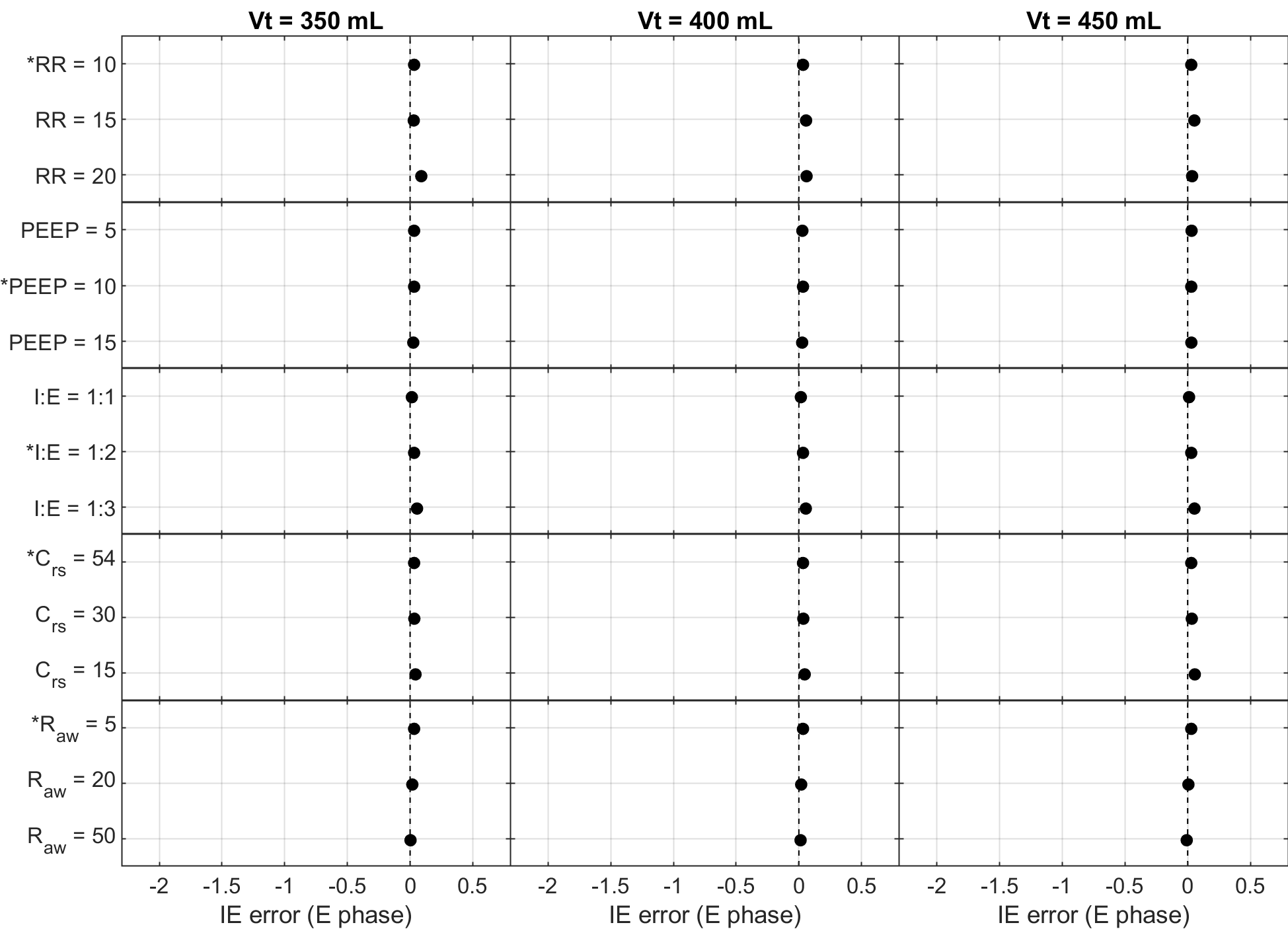

Supplement: Supplementary file 3 [file Data_Sheet_2.ZIP › Accuracy of Controls Systems/01_Data Storage/05_Accuracy figures/Hamilton T1_v2_IE_error_300dpi.tif]

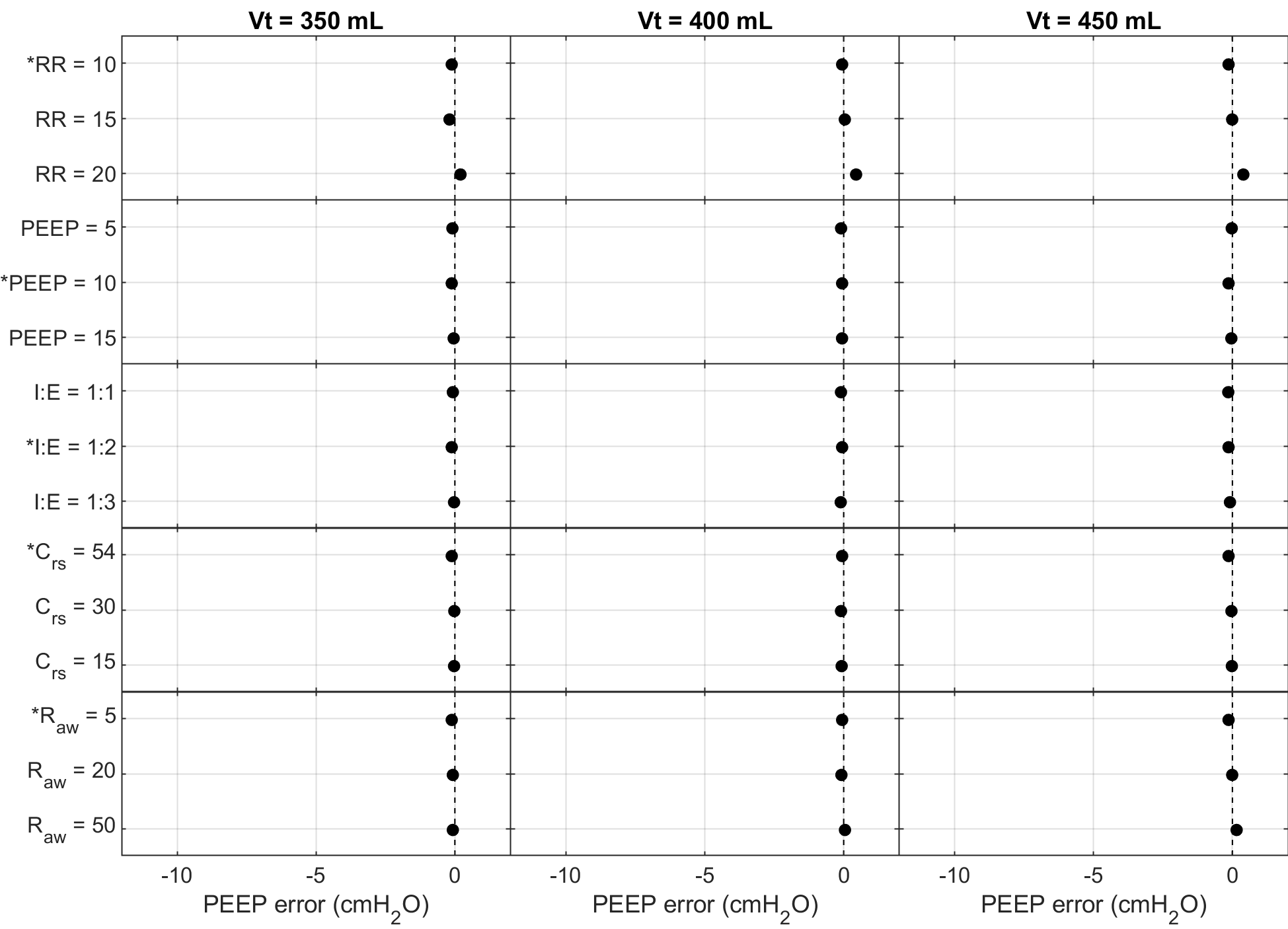

Supplement: Supplementary file 3 [file Data_Sheet_2.ZIP › Accuracy of Controls Systems/01_Data Storage/05_Accuracy figures/Hamilton T1_v2_PEEP_error_300dpi.tif]

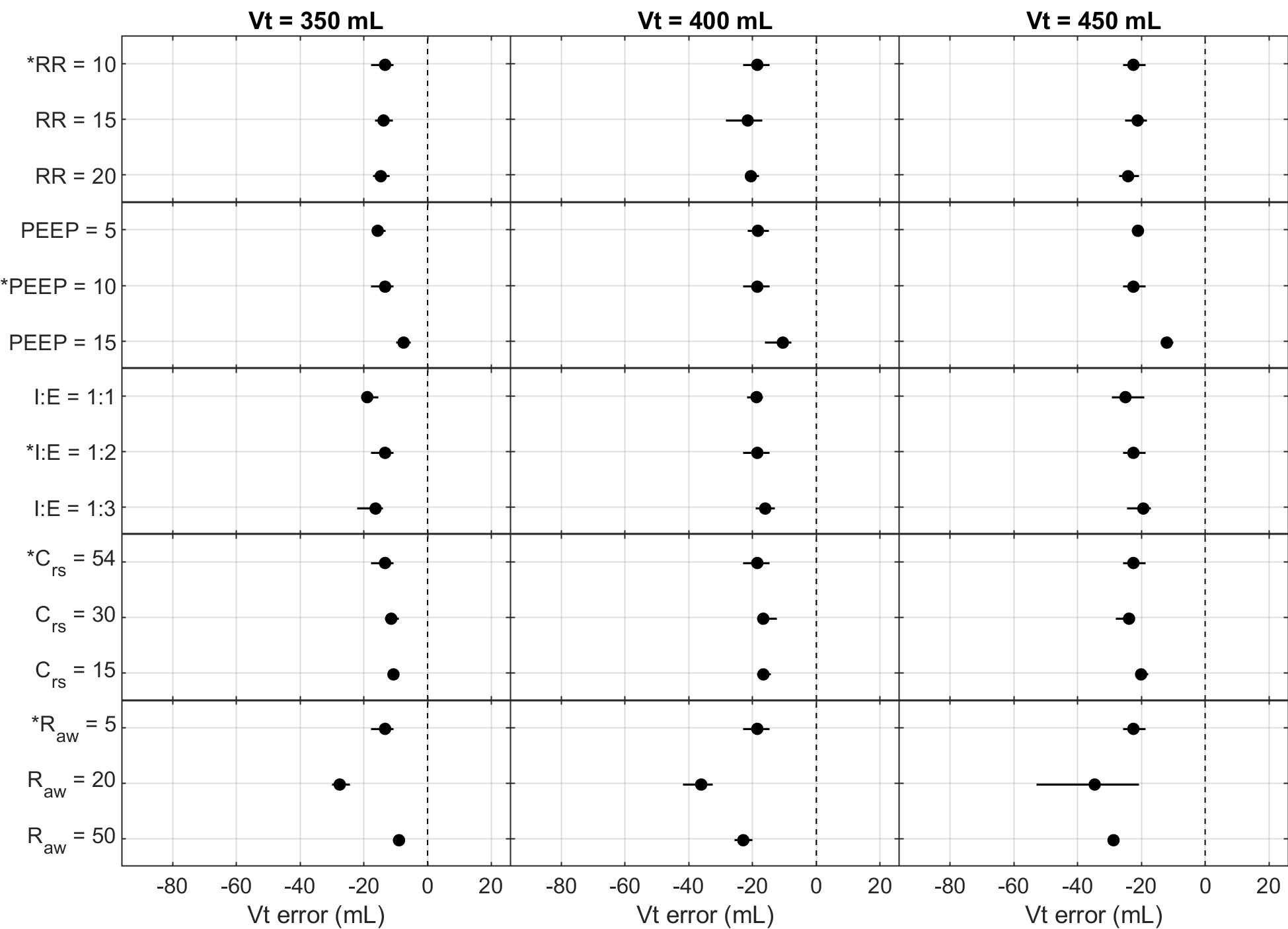

Supplement: Supplementary file 3 [file Data_Sheet_2.ZIP › Accuracy of Controls Systems/01_Data Storage/05_Accuracy figures/Hamilton T1_v2_Vt_error_300dpi.tif]

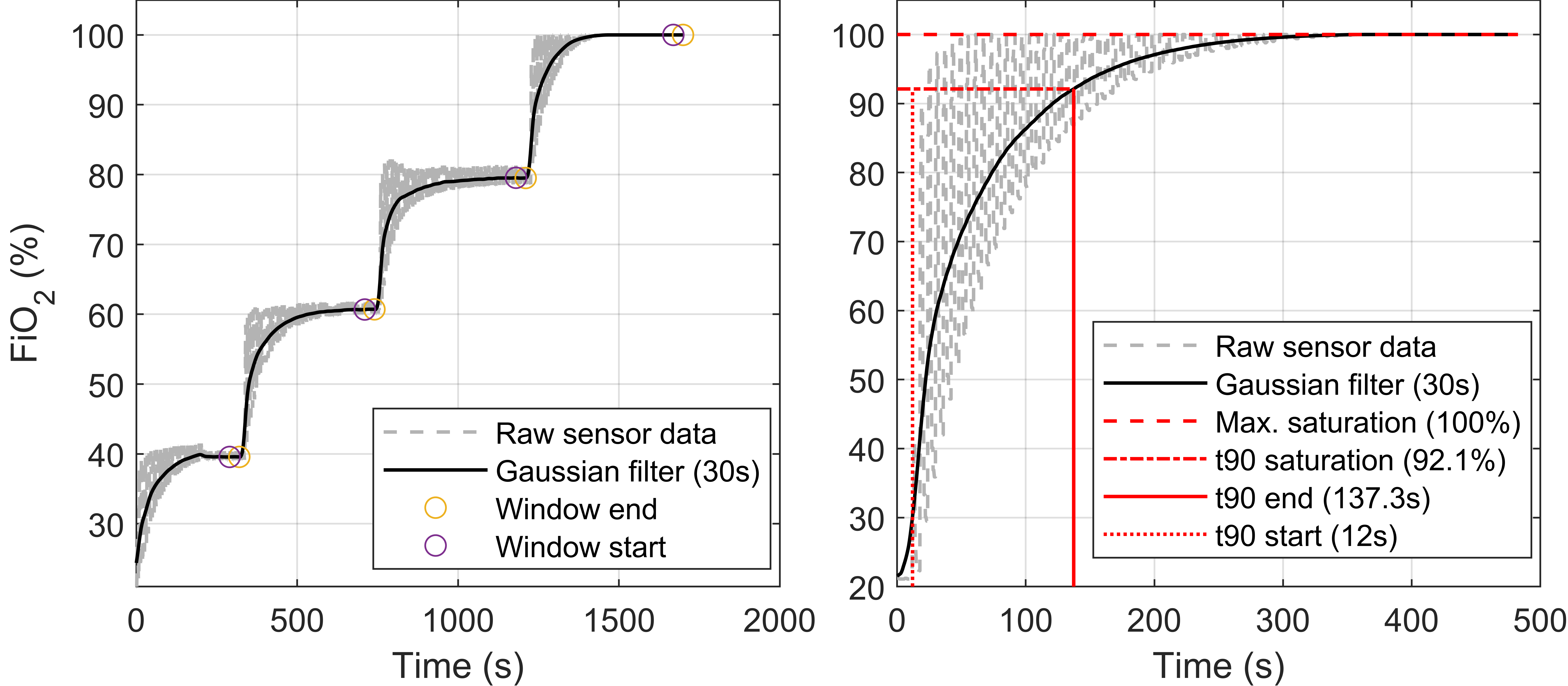

Supplement: Supplementary file 4 [file Data_Sheet_3.ZIP › O2 Evaluation/01_Data Storage/02_O2 plots/Hamilton T1_v1-HP_O2_1200dpi.tif]

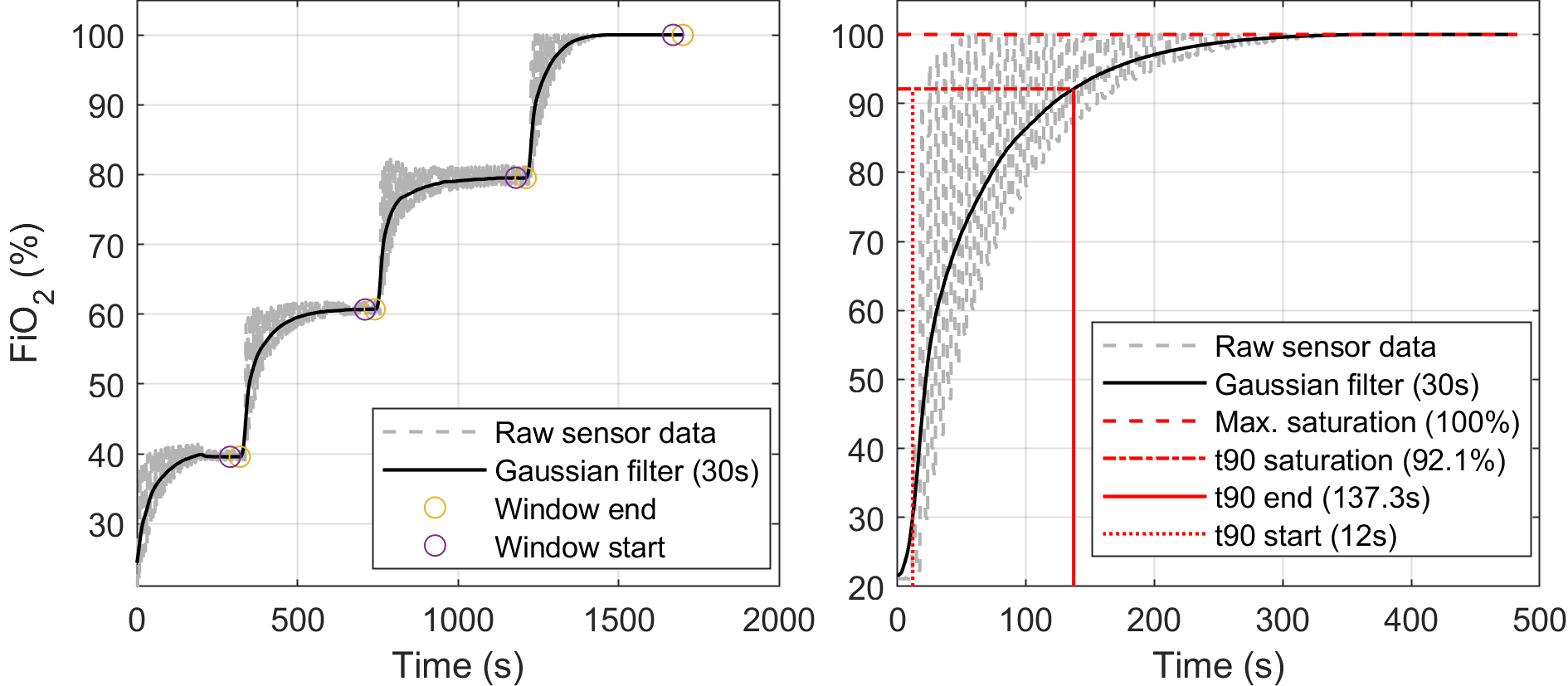

Supplement: Supplementary file 4 [file Data_Sheet_3.ZIP › O2 Evaluation/01_Data Storage/02_O2 plots/Hamilton T1_v1-HP_O2_300dpi.tif]

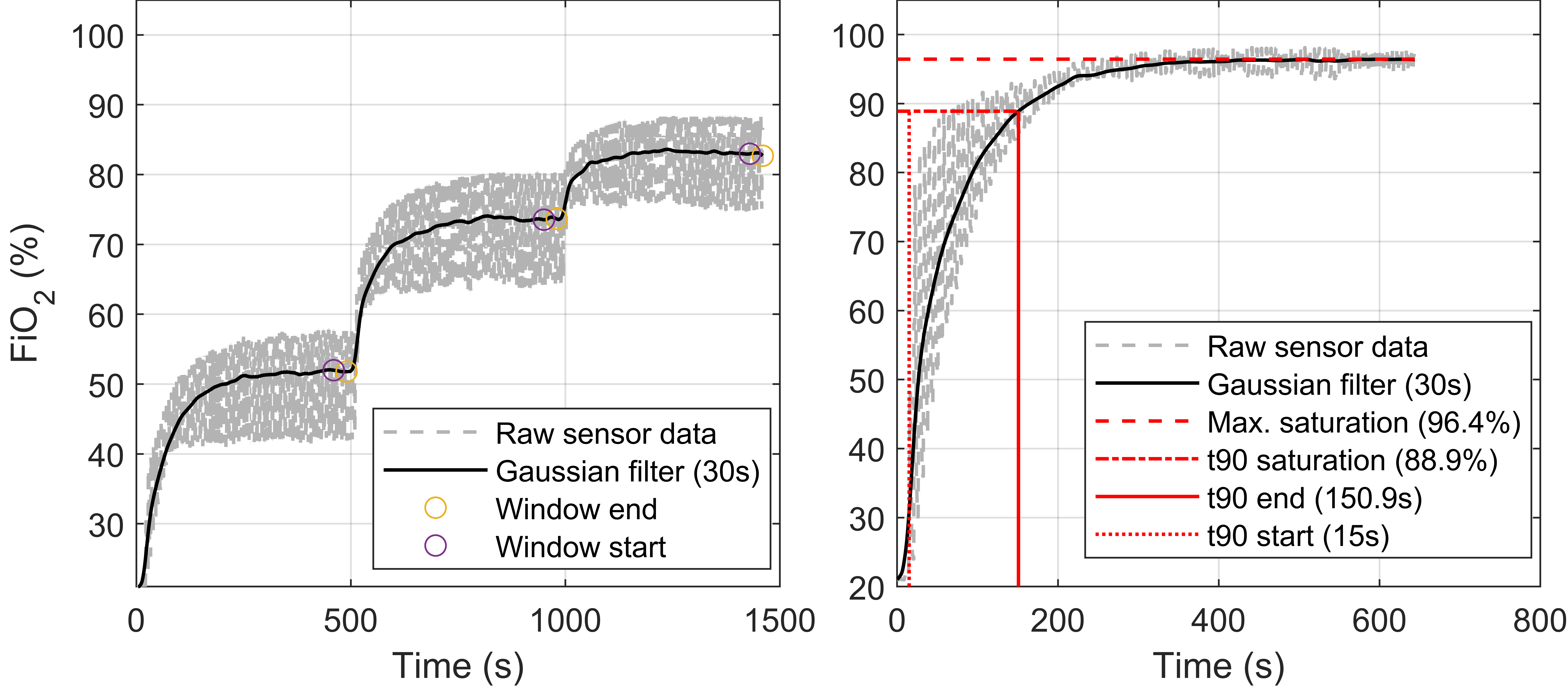

Supplement: Supplementary file 4 [file Data_Sheet_3.ZIP › O2 Evaluation/01_Data Storage/02_O2 plots/Hamilton T1_v1-LP_O2_1200dpi.tif]

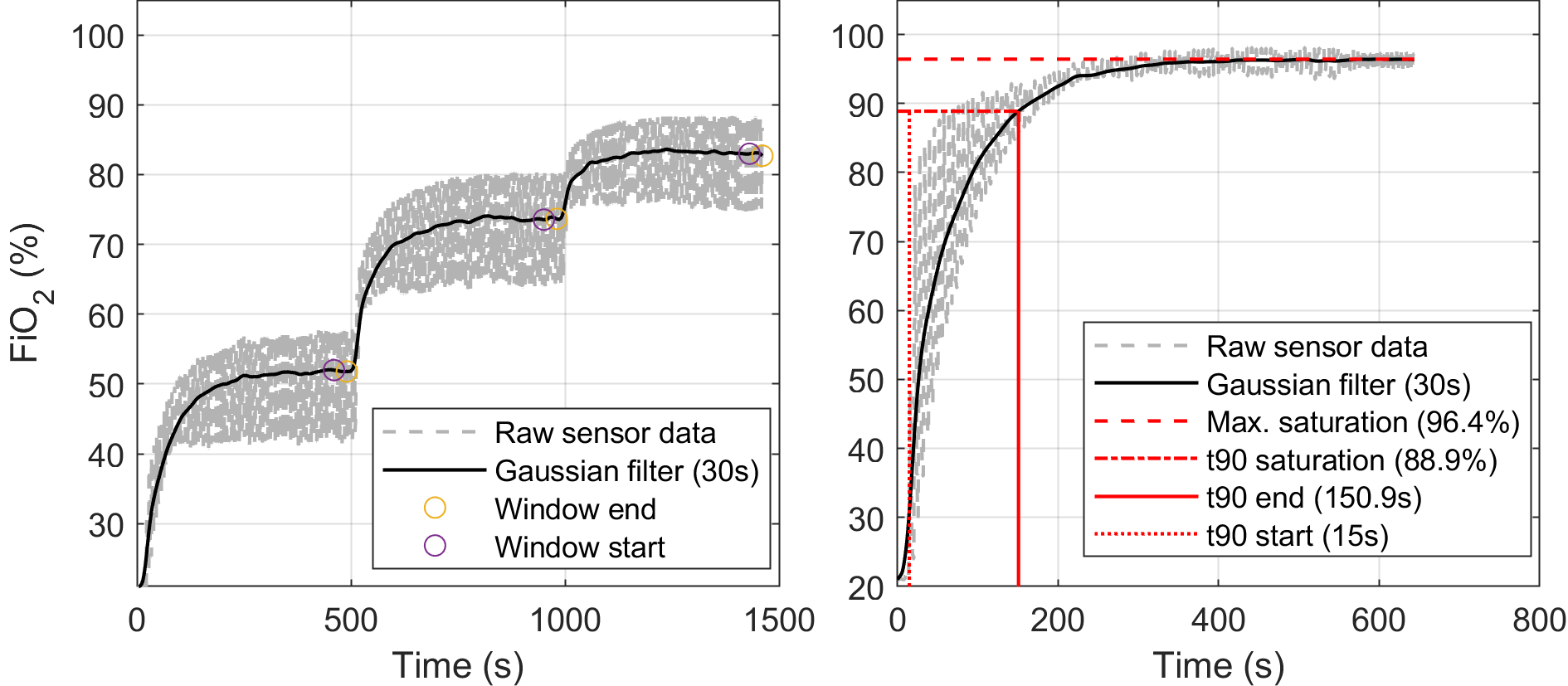

Supplement: Supplementary file 4 [file Data_Sheet_3.ZIP › O2 Evaluation/01_Data Storage/02_O2 plots/Hamilton T1_v1-LP_O2_300dpi.tif]
